# Prenylflavonoids isolated from *Epimedii Herba* show inhibition activity against advanced glycation end-products

**DOI:** 10.3389/fchem.2024.1407934

**Published:** 2024-05-31

**Authors:** Keisuke Nakashima, Hiroyuki Miyashita, Hitoshi Yoshimitsu, Yukio Fujiwara, Ryoji Nagai, Tsuyoshi Ikeda

**Affiliations:** ^1^ Faculty of Pharmaceutical Sciences, Sojo University, Kumamoto, Japan; ^2^ Department of Cell Pathology, Graduate School of Medical Sciences, Faculty of Life Sciences, Kumamoto University, Kumamoto, Japan; ^3^ Department of Food and Life Science, School of Agriculture, Tokai University, Kumamoto, Japan

**Keywords:** *Epimedii Herba*, prenylflavonoid, advanced glycation end products, N ε -(carboxymethyl)lysine, N ω -(carboxymethyl) arginine

## Abstract

**Introduction:** As inhibitors of advanced glycation end products (AGEs), such as pyridoxamine, significantly inhibit the development of retinopathy and neuropathy in rats with streptozotocin-induced diabetes, treatment with AGE inhibitors is believed to be a potential strategy for the prevention of aging, age-related diseases, and lifestyle-related diseases, including diabetic complications. In the present study, the MeOH extract of *Epimedii Herba* (EH; aerial parts of *Epimedium* spp.) was found to inhibit the formation of *N* ^*ε*^-(carboxymethyl)lysine (CML) and *N* ^*ω*^-(carboxymethyl) arginine (CMA) during the incubation of collagen-derived gelatin with ribose.

**Materials and methods:** EH was purchased from Uchida Wakan-yaku Co., and a MeOH extract was prepared. Several steps of column chromatography purified the extract. Each fraction was tested for inhibitory activity by ELISA using monoclonal antibodies for CML and CMA.

**Results:** After activity-guided fractionation and purification by column chromatography, three new prenylflavonoids [named Koreanoside L (**1**), Koreanoside E1 (**2**), and Koreanoside E2 (**3**)] and 40 known compounds (**4**–**43**) were isolated from EH, and their inhibitory effects against CML and CMA formation were tested. Among these, epimedokoreanin B (**8**), epimedonin E (**21**), epicornunin B (**22**), and epicornunin F (**24**) inhibited the formation of both CML and CMA, with epimedokoreanin B (**8**) having the most potent inhibitory effect among the isolated compounds. To obtain the structure–activity relationships of **8**, the phenolic hydroxy groups of **8** were methylated by trimethylsilyl-diazomethane to afford the partially and completely methylated compounds of **8**. Prenyl derivatives of propolis (artepillin C, baccharin, and drupanin) were used in the assay.

**Discussion:** As only **8** showed significant activity among these compounds, the catechol group of the B ring and the two prenyl groups attached to the flavanone skeleton were essential for activity. These data suggest that **8** could prevent the clinical complications of diabetes and age-related diseases by inhibiting AGEs.

## 1 Introduction

In recent years, preventive medicine has begun to play an important role in the aging population globally. Inhibiting the formation of advanced glycation end products (AGEs), which are involved in the progression of lifestyle-related diseases such as diabetic complications (Lin et al., 2011) and atherosclerosis (Torres et al., 2015), and aging-related diseases such as osteoporosis (Brandt et al., 2022) and Alzheimer’s disease (Barnard et al., 2014), is an effective method for the prevention of these diseases using natural products (Al-Musayeib et al., 2011; Harris et al., 2011; Aljohi et al., 2018; Tominaga et al., 2020). *N* ^ε^-(Carboxymethyl) lysine (CML), a major antigenic AGE structure, accumulates in several human and animal tissues during aging (Araki et al., 1992; Schleicher et al., 1997), and in patients with various diseases, including diabetic nephropathy (Vlassara et al., 1994; Rabbani and Thornalley, 2018) and encephalopathy. *N* ^ω^-(Carboxymethyl) arginine (CMA) is an acid-labile AGE structure discovered in the enzymatic hydrolysate of glycated collagen (Iijima et al., 2000). Collagen is an important protein that constitutes body tissues; however, it has been reported that when collagen becomes an AGE, it decreases both in strength and flexibility (Kitamura et al., 2021). CMA accumulation in tissue proteins may contribute to the pathophysiology of aging and age-related diseases (Mera et al., 2008; Kinoshita et al., 2019).

**FIGURE 1:**
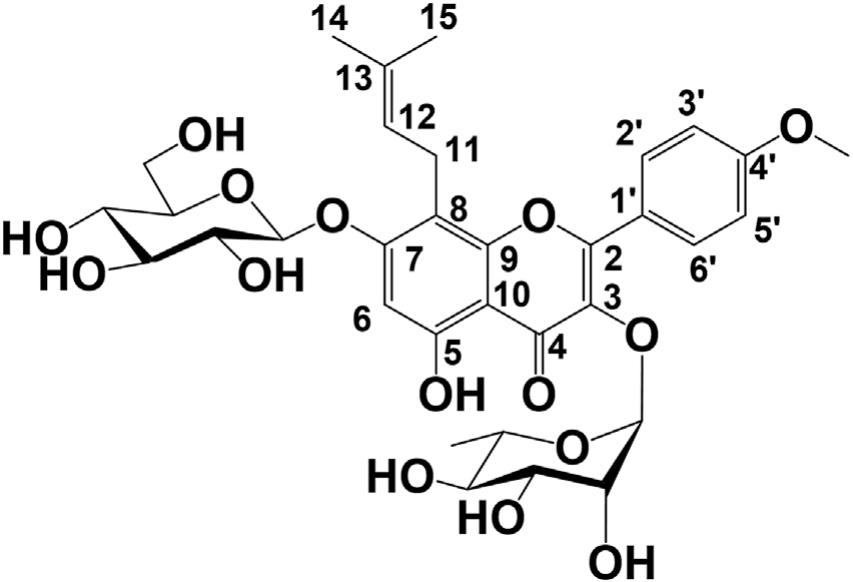
Structure of icariin, which is the main ingredient of EH.

*Epimedii Herba* (EH) has been used in traditional Chinese Medicine to treat erectile dysfunction, dysuria, waist and knee pain, infertility, and angina pectoris (Wu et al., 2003; Li C. et al., 2015; Chen et al., 2015; Qian et al., 2024). In Japan, the crude drugs listed in the Japanese Pharmacopeia and EH extracts are usually included in energy drinks for tonicity. Its main ingredients are prenylated flavonoids (Ma et al., 2011; Li et al., 2021), especially icariin (Figure 1), which suppresses nerve degeneration, improves cognitive function in neurological disorders (Guo et al., 2010), and has neuroprotective effects (Li et al., 2022). It has also been suggested that icariin inhibits AGE-derived neuropathy in PC12 cells (Zhao et al., 2019); RAGE might be a potential target for *Epimedium*’s anti-neuroinflammatory role in vascular dementia, which is an insight from network pharmacology and molecular simulation (Yuan et al., 2023), and extracts including icariin may reduce the risk of atherosclerosis by inhibiting the formation of AGEs on HDL (Kim and Shim, 2019). However, these studies were comparisons using readily available icariin, and the anti–glycation activity of the main body of EH was not considered due to the limited number of samples used in these evaluations. We have previously shown that some compounds isolated from EH have significant inhibitory effects on AGE formation (Nakashima et al., 2016). In this study, the isolation and purification of EH’s prenylflavonoid compounds was continued; 40 known compounds and three new compounds were isolated, and their chemical structures were determined. Based on the results of CML and CMA production inhibitory activity tests on 35 prenylflavonoids, of which quantities were available, the chemical structure characteristics necessary for the production inhibitory activity were summarized by synthesis of derivatives and comparison with related natural products and are reported.

## 2 Materials and methods

### 2.1 General experimental procedures

The optical rotation was measured using a P-1020 polarimeter (JASCO Co. Ltd., Tokyo, Japan). ^1^H- and ^13^C-NMR spectra were measured in pyridine-*d*_*5*_ and chloroform-*d* using a JEOL ECA 500 NMR spectrometer (JEOL Ltd., Tokyo, Japan) at 500 MHz and 125 MHz, respectively. The chemical shift (*δ*) was reported in parts per million (ppm). The *J* value was reported in Hz, using either pyridine-*d*_*5*_ as an internal standard for ^1^H NMR (7.20 ppm) and ^13^C NMR (123.5 ppm) or chloroform-*d* as an internal standard for ^1^H NMR (7.26 ppm) and ^13^C NMR (77.0 ppm). High-resolution electrospray ionization mass spectrometry (HRESIMS) spectra were recorded with a JMS-T100LP spectrometer (JEOL Ltd.). IR spectra were recorded using Jasco FT/IR-4200 spectrophotometer (JASCO Co. Ltd.). Preparative HPLC was performed on a Shimadzu HPLC system equipped with an LC-20AT pump (Shimazu Co. Ltd., Kyoto, Japan), JASCO 830-RI detector (JASCO), OR-2090 Plus chiral detector (JASCO), and Sugai U-620 column heater (Sugai Chemie Inc., Wakayama, Japan); COSMOSIL 5C_18_ AR-II, COSMOSIL π NAP (5 μm, ϕ10 × 250 mm, Nacalai Tesque Inc., Kyoto, Japan), and Atlantis Prep T3, SunFire Prep C_18_, X-Bridge Prep C_18_ (5 μm, ϕ10 × 250 mm, Waters Co., Milford, MA, United States) columns at a flow rate of 2.0 mL/min; and Triart PFP and Triart Phenyl (5 μm, ϕ4.6 × 250 mm, YMC Co. Ltd., Kyoto, Japan) columns at a flow rate of 1.0 mL/min, with each column temperature at 40 °C. For the analysis of sugar moieties, HPLC was performed on the Shodex RS-Pak DC-613 (5 μm, ϕ6.0 × 150 mm, Resonac Corp., Tokyo, Japan) column at a flow rate of 1.0 mL/min and a column temperature of 80°C. TLC was performed using pre-coated silica gel 60 F_254_ plates (Merck Ltd., Frankfurt, Germany). Detection was achieved by spraying the plates with 10% H_2_SO_4_ followed by heating. Column chromatography was carried out on MCI gel CHP20P (Mitsubishi Chemical Co., Tokyo, Japan), Sephadex LH-20 (GE Healthcare Bioscience Co., Uppsala, Sweden), μ-Bonda Pak C_18_ (ϕ25 × 200 mm, Waters Co.), silica gel 60 columns (230–400 mesh, Merck Ltd.), and Amberlite MB-3 (Organo Co., Tokyo, Japan).

### 2.2 Plant material

The aerial parts of *Epimedium* spp. were purchased from Uchida Wakan-yaku Co., Ltd. (Tokyo, Japan), inspected by Uchida Wakan-yaku, and certified as *Epimedii Herba* (EH; lot number: C1S1504) according to the specifications of the Japanese Pharmacopeia. A voucher specimen was deposited in the herbarium of the Faculty of Pharmaceutical Sciences, Sojo University (SJU1103).

### 2.3 Extraction and isolation

EH (3.0 kg) was extracted twice with MeOH by sonication for 6 h (30 min × 12) at 25 °C. The extract was concentrated under reduced pressure to obtain a residue (485 g). The residue was partitioned between *n*-hexane and 80% MeOH, after which the 80% MeOH layer was concentrated to yield a residue (408 g), which was loaded onto an MCI-gel CHP20P column and eluted with an H_2_O–MeOH gradient (0, 50, and 100% MeOH) to yield three fractions (frs. 1–3). Fr. 3 (65.0 g) was further applied to the MCI gel CHP20P column and eluted with an H_2_O–MeOH gradient (40, 50, 60, 70, 80, 90, and 100% MeOH) to yield eight fractions (frs. 3-1 to 3-8). Fr. 3-4 (9.0 g) was loaded onto a Sephadex LH-20 column (eluted with MeOH) to yield five fractions (frs. 3-4-1 to 3-4-5). A portion of fr. 3-4-3 (500 mg) was loaded for μ-Bonda Pak C_18_ column chromatography and eluted with an H_2_O–MeOH gradient (50, 60, 70, 80, 90% MeOH) to give six fractions (frs. 3-4-3-1 to 3-4-3-6). Fr. 3-4-3-3 (41.4 mg) was subjected to SiO_2_ (CHCl_3_: MeOH: H_2_O = 20:1:0 to 8:2:0.2 (*v/v*)) to yield six fractions (frs. 3-4-3-3-1 to 3-4-3-3-6). Fr. 3-4-3-3-3 (16.3 mg) was subjected to preparative HPLC [Atlantis Prep. T3 C_18_ (eluted with 70% MeOH)] to obtain compounds **2** (5.1 mg) and **3** (5.5 mg). Fr. 3-4-4 (660 mg) was subjected to Sephadex LH-20 chromatography (eluted with MeOH) to yield five fractions (frs. 3-4-4-1 to 3-4-4-5). Fr. 3-4-4-2 (285.9 mg) was subjected to μ-Bonda Pak C_18_ column chromatography and eluted with an H_2_O–MeOH gradient (60, 70, 80, 90% MeOH) to give nine fractions (frs. 3-4-4-2-1 to 3-4-4-2-9). Fr. 3-4-4-2-8 (3.0 mg) was also purified via preparative HPLC [X-Bridge Prep C_18_ (eluted with 80% MeOH)] to yield compound **1** (2.5 mg). The detailed isolation procedures for the other known compounds (**4**–**43**) is described in the Supplementary Material.

**Koreanoside L** (**1**): Yellow amorphous powder; *Rf* value 0.33 (solvent CHCl_3_: MeOH: H_2_O = 9:1:0.1); [*α*]_D_ −204 (*c* = 0.15, MeOH); ^1^H and ^13^C NMR (pyridine-*d*_*5*_, 500, and 125 MHz) data in Table 1; Positive ESIMS: *m/z* 533 [M + Na]^+^; HRESIMS 533.1461 [M + Na]^+^ (calculated for C_27_H_26_NaO_10_: 533.1424). IR (KBr) ν_max_ 3420, 2926, 1660, 1598, 1258 cm^-1^

**TABLE 1:** ^1^H and^13^C-NMR data for compound **1**-**3** in pyridine-*d*_*5*_.

Compound	1		2		3	
position	*δ*_C_	*δ*_H_ (*J* in Hz)	*δ*_C_	*δ*_H_ (*J* in Hz)	*δ*_C_	*d*_H_ (*J* in Hz)
2	157.7		156.5		156.8	
3	136.6		130.5		133.7	
4	179.7		178.8		179.4	
5	159.1		160.3		160.3	
6	94.9	6.84, 1H, s	99.5	6.81, 1H, s	99.1	6.84, 1H, s
7	159.1		164.0		164.0	
8	110.7		105.1		105.1	
9	156.9		155.2		155.3	
10	108.1		105.2		105.2	
11	100.5	7.27, 1H, s	30.3	3.43, 2H, m	30.3	3.44, 2H, m
12	156.9		75.1	4.92, 1H, dd, (5.7, 12.3)	74.9	4.92, 1H, t, (4.3)
13	132.7		104.8		105.0	
14	113.7	5.29, 5.89, each 1H, s	109.9	5.24, 4.89, each 1H, s	109.9	5.21, 4.87, each 1H, s
15	19.1	2.17, 3H, s	18.1	2.00, 3H, s	19.9	2.00, 3H, s
1′	122.9		122.3		122.3	
2′, 6′	131.4	8.29, 2H, d, (8.6)	130.8	8.20, 2H, d, (8.6)	131.6	8.28, 2H, d, (8.6)
3′, 5′	114.8	7.27, 2H, d, (8.6)	114.2	7.07, 2H, d, (8.6)	114.2	7.11, 2H, d, (8.6)
4′	162.0		161.8		161.8	
4′-OMe	55.7	3.82, 3H, s	55.1	3.67, 3H, s	55.1	3.67, 3H, s
rha-1	103.8	6.23, 1H, d, (1.8)	103.6	6.16, 1H, br s	103.1	6.25, 1H, br s
rha-2	72.1	5.12, 1H, t, (1.8)	71.5	5.04, 1H, d, (2.9)	71.4	5.06, 1H, d, (1.8)
rha-3	72.1	4.63, 1H, dd, (3.5, 9.7)	71.7	4.56, 1H, dd, (3.5, 9.8)	71.6	4.55, 1H, dd, (3.2, 9.2)
rha-4	72.8	4.32, 1H, t, (9.7)	72.0	4.26, 1H, t, (9.8)	72.0	4.23, 1H, t, (9.2)
rha-5	71.7	4.09, 1H, m	72.7	4.16, 1H, m	72.6	3.95, 1H, m
rha-6	18.2	1.41, 3H, d, (6.3)	18.0	1.39, 3H, d, (6.3)	17.8	1.30, 3H, d, (6.3)

**Koreanoside E1** (**2**): Yellow amorphous powder; *Rf* value 0.56 (solvent CHCl_3_: MeOH: H_2_O = 8:2:0.2); [*α*]_D_ −92.1 (*c* = 0.27, MeOH); ^1^H and ^13^C NMR (pyridine-*d*_*5*_, 500 and 125 MHz) data in Table 1; Negative ESIMS: *m/z* 529 [M-H]^-^; HRESIMS 529.1726 [M-H]^-^ (calculated for C_27_H_29_O_11_: 529.1710). IR (KBr) ν_max_ 3567, 2925, 1654, 1179 cm^-1^

**Koreanoside E2** (**3**): Yellow amorphous powder; *Rf* value 0.56 (solvent CHCl_3_: MeOH: H_2_O = 8:2:0.2); [*α*]_D_ −48.5 (*c* = 0.15, MeOH); ^1^H and ^13^C NMR (pyridine-*d*_*5*_, 500 and 125 MHz) data in Table 1; Negative ESIMS: *m/z* 529 [M-H]^-^; HRESIMS 529.1726 [M-H]^-^ (calculated for C_27_H_29_O_11_: 529.1710). IR (KBr) ν_max_ 3567, 2924, 1610, 1259, 1180 cm^-1^

### 2.4 Acid hydrolysis of compound 1

Compound **1** (1.0 mg) was hydrolyzed with 2 M HCl: dioxane = 1:1 solvent (1 mL) at 95°C in a pear-shaped flask for 1.5 h and H_2_O (2 mL) was added to the mixture, and it was evaporated to dryness under vacuum to obtain a residue. The residue was loaded onto Amberlite MB-3 (ϕ15 × 40 mm), eluted with H_2_O, subjected to an MCI gel CHP20P (ϕ15 × 40 mm), and the water elute was evaporated to dryness under vacuum. The residue was dissolved in CH_3_CN: H_2_O = 3:1 solution (20 μL) and analyzed by HPLC [Shodex RS-Pak DC-613 (ϕ6.0 × 150 mm, eluted with CH_3_CN: H_2_O = 3:1), flow rate 1.0 mL/min, 70 °C] connected to an optical rotatory detector. Then, by comparing the retention time and polarity of the standard [l-rhamnose: *t*_*R*_ = 4.5 min (−)], the constituent sugars of compound **1** was identified as l-rhamnose [*t*_*R*_ = 4.5 min (−)].

### 2.5 Enzymatic hydrolysis of compounds 2 and 3

Compounds **2** and **3** (2.5 mg each) were dissolved in DMSO (40 μL) and mixed with PBS (pH 6.2; 360 μL). Naringinase (10 mg; Sigma-Aldrich Corp., Saint Louis, MO, United States) was added to the mixtures and shaken at 40 °C, 120 rpm, for 24 h. The mixtures were centrifuged to remove the supernatant and the precipitate was dissolved in a small amount of pyridine and loaded onto SiO_2_ [ϕ10 × 130 mm, CHCl_3_: MeOH: H_2_O = 9:1:0.1 (*v/v*)] to obtain aglycones **2a** (1.9 mg, 73% yield) and **3a** (2.0 mg, 75% yield), respectively. HPLC analyzed each supernatant from compounds **2** and **3,** coupled with an optical rotatory detector, to identify l-rhamnose [*tR* = 4.5 min (−)].

**Compound 2a**: *Rf* value: 0.78 (solvent CHCl_3_: MeOH: H_2_O = 9:1:0.1), [*α*]_D_ +5.5 (*c* = 0.038), ^1^H-NMR (500 MHz, CDCl_3_) [*δ*_H_ 8.12 (2H, d, *J* = 9.1 Hz, H-2′, 6′), 7.02 (2H, d, *J* = 9.1 Hz, H-3′, 5′), 6.44 (1H, s, H-6), 5.09, 4.95 (each 1H, br s, H-14a,b), 4.47 (1H, br d, *J* = 8.6 Hz, H-12), 3.89 (3H, s, 4′-OMe), 3.34 (1H, br d, *J* = 15.4 Hz, H-11a), 3.02 (1H, dd, *J* = 8.6, 15.4 Hz, H-11b), 1.87 (3H, s, H-15)].

**Compound 3a**: *Rf* value: 0.78 (solvent CHCl_3_: MeOH: H_2_O = 9:1:0.1), [*α*]_D_ −14.0 (*c* = 0.038), ^1^H-NMR (500 MHz, CDCl_3_) [*δ*_H_ 8.17 (2H, d, *J* = 6.9 Hz, H-2′, 6′), 7.03 (2H, d, *J* = 6.9 Hz, H-3′, 5′), 5.04, 4.88 (each 1H, br s, H-14a,b), 4.39 (1H, br d, *J* = 8.6 Hz, H-12), 3.90 (3H, s, 4′-OMe), 3.24 (1H, dd, *J* = 2.9, 14.9 Hz, H-11a), 3.04 (1H, dd, *J* = 9.1, 14.9 Hz, H-11b), 1.85 (3H, s, H-15)].

### 2.6 Synthesis of MTPA esters of compounds 2a and 3a

Compound **2a** (1.5 mg) was placed in a pear-shaped flask and dissolved in MeOH (200 μL); 2 M trimethylsilyl (TMS)-diazomethane 800 μL (67 eq) at 0 °C was added, and the reaction was stirred for 1 h at 20 °C. The reactant was purified using SiO_2_ [ϕ10 × 70 mm, CHCl_3_: MeOH = 50:1 (*v/v*)] to obtain compound **2b** (1.0 mg, 60% yield). Next, **2b** (1.0 mg, 2.34 μmol) was placed in a pear-shaped flask sealed with nitrogen purge, and CH_2_Cl_2_ (200 μL), (*R*)-(−)-α-methoxy-α-(trifluoromethyl)phenylacetyl chloride [(*R*)-(−)-MTPA-Cl, *ca*. 18% in dichloromethane, *ca*. 1.0 mol/L; TCI Reagents, Tokyo, Japan], was added to 70 μL (70 μmol, 30 eq) dimethylaminopyridine (DMPA), water-soluble carbodiimide (WSC), and triethylamine (TEA) and reacted for 1 h. The reactant was purified using SiO_2_ [ϕ10 × 100 mm, CHCl_3_: MeOH = 50:1 (*v/v*)] and HPLC [Triart PFP (ϕ4.6 × 250 mm, eluted with 85% MeOH)] to give **2b**-(*S*)-MTPA ester (**2b-*S***, 0.8 mg, 53% yield). Similarly, **2a** (1.6 mg) was reacted with the (*S*)-(+)-MTPA-Cl reagent to give **2b**-(*R*)-MTPA ester (**2b-*R***, 0.5 mg, 38% yield). Compound **3a** was methylated and purified using the same method as that used for **2a** to obtain **3b**. After that, **3b** was reacted with (*R*)-(−)-MTPA-Cl and (*S*)-(+)-MTPA-Cl in the above methods to give MTPA ester **3b-*S*** (1.5 mg, 47% yield) and **3b-*R*** (0.5 mg, 35% yield), respectively.

**Compound 2b-*S* (= compound 3b-*R*):** *Rf* value: 0.78 (solvent CHCl_3_: MeOH = 100:1), ^1^H-NMR (500 MHz, CDCl_3_) [*δ*_H_ 8.02 (2H, d, *J* = 9.1 Hz, H-2′, 6′), 6.97 (2H, d, *J* = 9.1 Hz, H-3′, 5′), 6.28 (1H, s, H-6), 5.79 (1H, br d, *J* = 6.3 Hz, H-12), 5.11, 5.01 (each 1H, br s, H-14a,b), 4.00, 3.89, 3.88, 3.86 (each 3H, s, 4’, 3, 5, 7-OMe), 3.40 (1H, dd, *J* = 10.3, 15.3 Hz, H-11a), 3.01 (1H, dd, *J* = 4.0, 15.3 Hz, H-11b), 1.89 (3H, s, H-15)]

**Compound 2b-*R* (= compound 3b-*S*):** *Rf* value: 0.78 (solvent CHCl_3_: MeOH = 100:1), ^1^H-NMR (500 MHz, CDCl_3_) [*δ*_H_ 8.01 (2H, d, *J* = 7.4 Hz, H-2′, 6′), 6.88 (2H, d, *J* = 7.4Hz, H-3′, 5′), 6.37 (1H, s, H-6), 5.79 (1H, dd, *J* = 4.0, 9.7 Hz, H-12), 4.99, 4.96 (each 1H, br s, H-14a,b), 4.02, 3.92, 3.89, 3.88 (each 3H, s, 4’, 3, 5, 7-OMe), 3.43 (1H, dd, *J* = 10.3, 13.8 Hz, H-11a), 3.03 (1H, dd, *J* = 4.0, 14.3 Hz, H-11b), 1.74 (3H, s, H-15)]

### 2.7 Partial methylation of phenolic hydroxyl groups in epimedokoreanin B

Epimedokoreanin B (**EK-B**, isolated compound number **39**, 20 mg, 47.4 μmol) was placed in a pear flask and dissolved in MeOH (200 μL). Then, 500 μL (5 eq) of approximately 2 M TMS-diazomethane solution in diethyl ether (TCI Reagents, Tokyo, Japan) was added at 0 °C, and this mixture was allowed to react for 1 h at 20 °C. The reactants were purified using silica gel SiO_2_ [ϕ10 × 70 mm, eluted with hexane: acetone = 3:1 (*v/v*)] and HPLC [Triart PFP (ϕ4.6 × 250 mm, eluted with 100% MeOH)] to yield dimethoxy EK-B (1.6 mg, 11% yield), trimethoxy EK-B (2.6 mg, 25% yield), and tetramethoxy EK-B (5.5 mg, 19% yield), respectively.

**Dimethoxy EK-B (A):** *Rf* value: 0.60 (solvent hexane: acetone = 2:1), ^1^H-NMR (500 MHz, CDCl_3_) [*δ*_H_ 7.41 (1H, s, H-2′), 7.34 (1H, s, H-6′), 6.60 (1H, s, H-3), 6.42 (1H, s, H-6), 5.27 (1H, t, *J* = 6.4 Hz, H-12), 4.90 (1H, t, *J* = 6.3 Hz, H-2″), 3.94, 3.90 (each 3H, s, 4’, 7-OMe), 3.52 (2H, d, *J* = 8.3 Hz, H-11), 4.92 (2H, d, *J* = 7.5 Hz, H-1″), 1.84 (3H, s, H-15), 1.80 (3H, s, H-5″), 1.71 (3H, s, H-4”), 1.69 (3H, s, H-14)].

**Trimethoxy EK-B (B):** *Rf* value: 0.75 (solvent hexane: acetone = 2:1), ^1^H-NMR (500 MHz, CDCl_3_) [*δ*_H_ 7.41 (1H, s, H-2′), 7.34 (1H, s, H-6′), 6.60 (1H, s, H-3), 6.42 (1H, s, H-6), 5.27 (1H, t, *J* = 6.4 Hz, H-12), 4.90 (1H, t, *J* = 6.3 Hz, H-2″), 3.94, 3.91, 3.90 (each 3H, s, 3′, 4’, 7-OMe), 3.52 (2H, d, *J* = 8.3 Hz, H-11), 4.92 (2H, d, *J* = 7.5 Hz, H-1″), 1.84 (3H, s, H-15), 1.80 (3H, s, H-5″), 1.71 (3H, s, H-4”), 1.69 (3H, s, H-14)].

**Tetramethoxy EK-B (C):** *Rf* value: 0.20 (solvent hexane: acetone = 2:1), ^1^H-NMR (500 MHz, CDCl_3_) [*δ*_H_ 7.41 (1H, s, H-2′), 7.34 (1H, s, H-6′), 6.60 (1H, s, H-3), 6.42 (1H, s, H-6), 5.27 (1H, t, *J* = 6.4 Hz, H-12), 4.90 (1H, t, *J* = 6.3 Hz, H-2″), 3.94, 3.91, 3.90, 3.90 (each 3H, s, 3′, 4’, 5, 7-OMe), 3.52 (2H, d, *J* = 8.3 Hz, H-11), 4.92 (2H, d, *J* = 7.5 Hz, H-1″), 1.84 (3H, s, H-15), 1.80 (3H, s, H-5″), 1.71 (3H, s, H-4”), 1.69 (3H, s, H-14)].

### 2.8 Determination of the inhibitory effects of compounds on CML and CMA formations

Gelatin (2 mg/mL) and ribose (30 mM) were incubated with the tested compounds in PBS for CML and in 100 mM sodium phosphate buffer for CMA at 37 °C for 7 days, followed by the determination of CML and CMA formation using a noncompetitive enzyme-linked immunosorbent assay (ELISA).

### 2.9 ELISA

ELISA was performed as previously described (Sugawa et al., 2016). Briefly, each well of a 96-well microtiter plate was coated with 100 μL of the sample in PBS, blocked with 0.5% gelatin, and washed three times with PBS containing 0.05% Tween-20 (washing buffer). The wells were incubated with 100 μL of anti-CML antibody 6D12 (0.1 μg/mL) or anti-CMA antibody 3F5 (1.0 μg/mL) dissolved in washing buffer for 1 h. The wells were then washed three times with washing buffer and incubated with horseradish peroxidase-conjugated anti-mouse IgG antibodies, followed by incubation with 1,2-phenylenediamine dihydrochloride. The reaction was terminated by the addition of 100 μL of 1 M sulfuric acid and the absorbance at 492 nm was read by a micro-ELISA plate reader.

### 2.10 Statistics

All data are representative of two or three independent experiments. Data are expressed as mean (SD). The Mann–Whitney *U* test was used for two-group comparisons. Statistical significance was set at *p* < 0.05.

## 3 Result and discussion

### 3.1 Isolation and determination of new compounds (1–3) from EH

Following activity-guided fractionation of the three fractions prepared in a previous report (Nakashima et al., 2016) (frs. 1, 2, and 3), fr. Three was further separated by column chromatography combined with MCI gel CHP20P, Sephadex LH-20, μ-Bondapak C_18_, and preparative HPLC. Then, 43 candidate prenylated flavonoid derivatives, including new compounds (**1**–**3**), were isolated to test the inhibition of CML and CMA accumulation.

**FIGURE 2:**
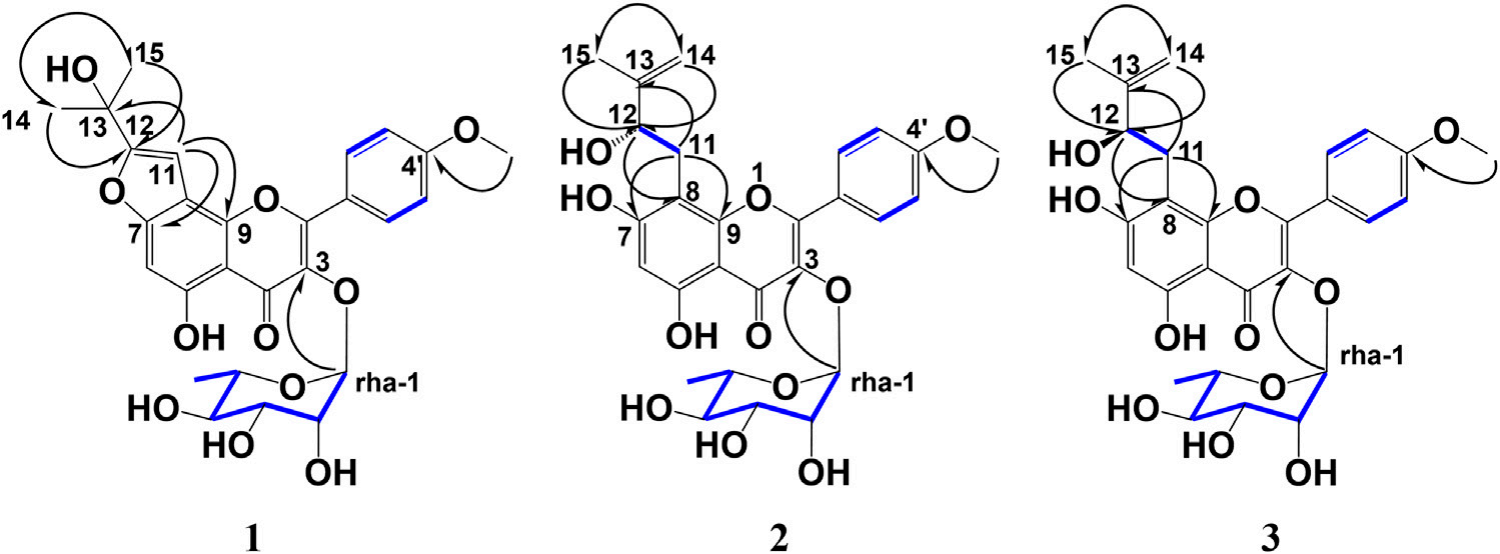
Key HMBC and ^1^H–^1^H COSY correlations of koreanoside L (**1**), koreanoside E1 (**2**), and koreanoside E2 (**3**).

Compound **1** was obtained as an amorphous yellow powder. The molecular formula (C_27_H_26_O_10_) was established based on ^1^H-, ^13^C-NMR, HRESIMS (*m/z* 533.1461 [M + Na]^+^, and calculated for C_27_H_26_O_10_Na: 533.1448). In the ^1^H-NMR data (Table 1), exo-methylene protons [*δ*_H_ 5.29, 5.89 (each 1H, s, H-14)], olefinic methyl protons [*δ*_H_ 2.17 (3H, s, H-15)], and a tri-substitute olefinic proton [*δ*_H_ 7.27 (1H, s, H-11)] suggested a 2-(prop-1-en-2-yl) furan moiety. In addition, two para-coupled aromatic protons [*δ*_H_ 8.29 (2H, d, *J* = 8.6 Hz, H-2′, 6′), 7.27 (2H, d, *J* = 8.6 Hz, H-3′,5′)], a methoxy group signal [*δ*_H_ 3.82 (3H, s)], an isolated anomeric proton signal [*δ*_H_ 6.23 (1H, d, *J* = 1.8 Hz, H-1 of rhamnose)], and a doublet methyl protons signal [*δ*_H_ 1.41 (3H, d, *J* = 6.3 Hz, H-6 of rhamnose)] were observed (Table 1). The ^13^C NMR and HMQC data indicated 27 carbon resonances (Table 1), corresponding to a flavone derivative with an exo-methylene group, 2-(prop-1-en-2-yl) furan, and deoxyhexose moiety. Compound **1** was acid-hydrolyzed, and the resulting sugar fraction was analyzed by HPLC connected to an optical rotatory detector, and the peak of L-(−)-rhamnose was observed. The heteronuclear multiple bond correlations (HMBCs) were found between H-11 (*δ*_H_ 7.27) and C-7 (*δ*_C_ 159.1), C-8 (*δ*_C_ 110.7), C-9, C-12 (*δ*_C_ 156.9), and C-13 (*δ*_C_ 132.7), as well as those between H-14 (*δ*_H_ 5.29, 5.89) and C-12 (*δ*_C_ 156.9), H-15 (*δ*_H_ 2.17) and C-12, C-13, and C-14 (*δ*_C_ 113.7). Thus, compound **1** confirmed that the 2-(prop-1-en-2-yl) furan moiety was connected to C-7 and C-8. Further, HMBCs were also found between the H-1 of rhamnose (*δ*_H_ 6.23) and C-3 (*δ*_C_ 136.6), the methoxy group signal (*δ*_H_ 3.82), and C-4’ (*δ*_C_ 162.0). Therefore, the rhamnose was connected at C-3, and the methoxy group was connected at C-4 (Figure 2). Thus, the structure of **one** was deduced as 3-*O*-α-L-rhamnopyranosyl-5-hydroxy-2-(4-methoxyphenyl)-8-(prop-1-en-2-yl)-2*H*-pyran-2-yl)oxy)-4*H*-furo [2,3-h]chromen-4-one, and named koreanoside L.

**FIGURE 3:**
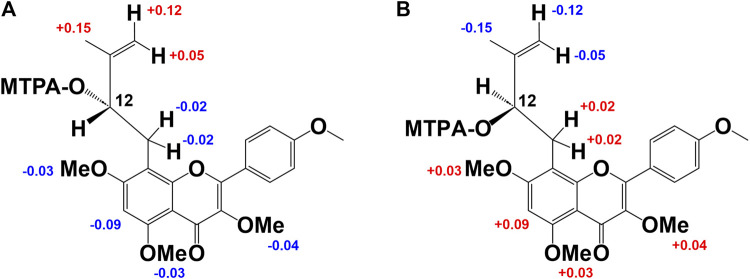
Modified Mosher’s method results of derivative from **2b** and **3b**. Difference of the ^1^H-NMR chemical shifts of **(A)**: **2b-*S*** and **2b-*R*** and **(B)**: **3b-*S*** and **3b-*R*** [Δ*δ* (in ppm) = *δ*_*S*_ - *δ*_*R*_].

Compound **2** was obtained as a yellow amorphous powder and the rotation value was [*α*]_D_ −92.1° (*c* = 0.27). The molecular formula (C_27_H_30_O_11_) of **two** was established based on ^1^H, ^13^C-NMR, and HRESIMS (*m/z* 529.1726 [M−H]^−^; it was calculated for C_27_H_29_O_11_: 529.1726). In the ^1^H-NMR spectrum (Table 1), compound **2** was found in an *O*-methyl group (*δ*_H_ 3.67, 3H, s), AA’BB’ coupling system [*δ*_H_ 8.20 (2H, d, *J* = 8.6 Hz, H-2′,6′), and *δ*_H_ 7.07 (2H, d, *J* = 8.6 Hz, H-3′, 5′)]; an aromatic H-atom singlet [*δ*_H_ 6.81 (1H, s, H-6)], a tertiary methyl group [*δ*_H_ 2.00 (3H, s, H-15)], exo-methylene protons [*δ*_H_ 5.24 and 4.89 (1H, each s, H-14a, b)], an *O*-bearing CH [*δ*_H_ 4.92 (dd, *J* = 5.7, 12.3 Hz, H-12)], and a methylene group [*δ*_H_ 3.43 (2H, m, H-11)] were observed. These results suggested the presence of a 3-methyl-but-3-en-2-ol group. In addition, the anomeric proton signal [*δ*_H_ 6.16 (1H, br s)] suggested the presence of a rhamnose moiety. ^13^C-NMR data indicated that it was a flavone derivative with a 3-methyl-but-3-en-2-ol group and rhamnose moiety. HMBCs were found between the anomeric proton (*δ*_H_ 6.16) and C-3 (*δ*_C_ 130.5), the methylene proton of the 3-methyl-but-3-en-2-ol group (*δ*_H_ 3.43) and C-7 (*δ*_C_ 164.0), C-9 (*δ*_C_ 155.2), and the hydroxy methine proton at H-12 (*δ*_H_ 4.92) and C-8 (*δ*_C_ 105.1), and the *O*-methyl proton (*δ*_H_ 3.67) and C-4’ (*δ*_C_ 161.8). Thus, the planar structure of compound **3** was determined as shown in Figure 2. While compound **3** has been reported as koreanoside E and the NMR data were in good agreement (Li X. et al., 2015), the absolute steric structure of C-12 was not discussed. To determine the absolute steric structure of C-12, a modified Mosher method was applied to compound **2** (Ohtani et al., 1991; Tabopda et al., 2008). At first, to cleave the rhamnose moiety, compound **3** was treated with enzymatic hydrolysis using naringinase, and an aglycone **2a** in 73% yield was obtained. The supernatant of the reactant was analyzed using HPLC connected to an optical rotatory detector, and the L-(−)-rhamnose peak was observed. **2a** was obtained as a yellow amorphous powder and the rotation value was [*α*]_D_ +5.5 (*c* = 0.038). The structure of **2a** was confirmed by ^1^H-NMR data. The phenolic hydroxyl group of **2a** was methylated with TMS-diazomethane. The reactant was purified using SiO_2_ and preparative HPLC to obtain methylated **2a** (compound **2b**) in 87% yield. Finally, compound **2b** was reacted with (*R*)-(−)-α-methoxy-alpha-(trifluoromethyl) phenylacetyl chloride [(*R*)-(−)-MTPA-Cl] and (*S*)-(+)-MTPA-Cl to give MTPA esters **2b-*S*** and **2b-*R*** in 53% and 38% yields, respectively. Both compounds were measured using ^1^H-NMR and adapted to the modified Mosher method. Δ*δ*_H_ of **2b-*S*** and **2b-*R*** were H-15 (+0.15), 14a, b (+0.12, +0.05); and H-11a, b (each −0.02), H-6 (−0.09), 7-OMe (−0.03), 5′-OMe (−0.03), and 3′-OMe (−0.04), respectively. It is evident that protons with >0 are located on the right side of the MTPA plane and those with <0 are on the left side (Figure 3A). Therefore, the absolute conformation C-12 of compound **2** was determined as ***S***. Compound **2** was assigned the trivial name, **koreanoside E1**.

Compound **3** was similar to compound **2** in the ^1^H-NMR spectrum, but its rotation value was different ([*α*]_D_ −48.5° (*c* = 0.36)). The ^13^C-NMR date of **three** and **two** were identical but C-3 (*δ*_C_ 130.5–133.7), C-4 (*δ*_C_ 178.8–179.4), and C-15 (*δ*_C_ 18.1–19.9) data were shifted lower in the field. Because of the possibility of stereoisomerism at the C-12 position, compound **3** was hydrolyzed by naringinase to get an aglycone **3a** in 75% yield. Compound **3a** was obtained as a yellow amorphous powder and the rotation value was [*α*]_D_ −14.0 (*c* = 0.038). The ^1^H-NMR of **3a** was identical to that of **2a**. The supernatant of reactant was also analyzed by HPLC connected to an optical rotatory detector, and the peak of L-(−)-rhamnose was observed. To adapt the modified Mosher method, the phenolic hydroxyl groups of **3a** were methylated and purified to yield **3b**. Subsequently, **3b** was reacted with (*R*)-(−)-MTPA-Cl to give MTPA ester **3b-*S*** in 47% yield. The ^1^H-NMR data of **3b-*S*** was superimposable to that of **2b-*R***, so that it was applied to the advanced Mosher method because the ^1^H-NMR signals of **2b-*S*** were the same as those of **3b-*R***. As a result, the distribution of chemical shifts, as shown in Figure 3B, was observed, and the absolute conformation at C-12 of compound **3** was determined as ***R***. Compound **3** was given the trivial name **koreanoside E2**. This indicated that compound **3** was a diastereomer of compound **2** because of the rhamnose bond to C-3, which could be separated by HPLC.

The known compounds (**4**–**43**) were isolated by column chromatography, as described in the Supplementary Material, and were identified as prenylated flavonoids or flavanones (Supplementary Figures S1–S6). Compound **4** was identified as koreanoside I, which was obtained from *Epimedium koreanum* as an antipulmonary fibrosis compound (Zhao et al., 2022). Compound **18** was icariin, which is the main component of EH. Compounds **7**, **8**, and **9** were related to icariin, and their terminal sugar moieties attached to the C-2 of rhamnose were glucose, rhamnose, and xylose, respectively. These were called epimedins A (**7**), B (**8**), and C (**9**) (Oshima et al., 1987; Mizuno et al., 1988). Compound **10** was identified as epimedin I (**10**) (Sun et al., 1998; Zhao et al., 2007), which had glucose attached to the C-3 of rhamnose in icariin. Compounds **11**, **12**, **13**, and **14** were diacetyl compound **10** with a terminal glucose moiety. Compounds **11**, **12**, and **13** were attached to two acetyl groups at C-2, 6; C-3, 6; and C-4, six of glucose, respectively. Compound **14** was attached to an acetyl group at C-6 of glucose, and the glucose of compound **28** was substituted with a hydroxy group at C-7 in compound **14**. These have been reported to be epimedin K (**11**), epimedin L (**12**), caohuoside B (**13**), epimedokoreanoside I (**14**), and korepimedoside A (**28**), respectively (Zhao et al., 2007). Compounds **6, 18, 19,** and **20** were diglycosides with hydroxyl groups at C-3 and prenyl groups at C-8. They were identified as cuhuoside (**6**) (Zhao et al., 2007), icariin (**18**) (Xia et al., 2010), sagittatoside A (**19**) (Mizuno et al., 1988), and 2″-*O*-rhamnosyl icariside II (**20**) (Zhang et al., 2007), respectively. Compounds **25**, **26**, and **27** had two acetyl groups on the terminal glucose of korepimedoside A (**28**). The positions of the acetyl groups were C-2, 6; C-3, 6; and C-4, 6, respectively. They were reported as korepimeosides A (**25**) and B (**26**) (Li et al., 2016), and epimedigrandioside A (**27**) (Zulfiqar et al., 2017), respectively. Compounds **5**, **16**, **17**, **21**, **22**, **23**, **24**, and **30** were monoglycosides with rhamnose attached to the C-3 of the C-ring or glucose attached to the C-7 of the A-ring. They were identified as koreanoside F (**16**), G (**17**) (Choi et al., 2019), epimedoside C (**5**), pherodendroside (**21**) (Li et al., 1998), caohuoside C (**22**) (Zhao et al., 2007), icariside II (**23**) (Xia et al., 2010), icarisoside A (**24**) (Li et al., 2016), and icariside I (**30**) (Xia et al., 2010), respectively. The other isolated compounds were prenylated aglycones of flavonoids or flavanones. They were identified as epimedokoreanin C (**15**) (Li et al., 1994), 8-prenyl kaempferol (**29**), 8-prenyl luteolin (**31**) (Dong et al., 2007), epicornunin B (**32**), F (**33**) (Pang et al., 2018), gaocaonin E (**34**), euchrestaflavanone A (**35**) (Nakahara et al., 2003)[48], epimedonin C (**36**) (Jin et al., 2014), 8,5′-diprenyl apigenin (**37**), broussonol D (**38**) (Zhang et al., 2001), epimedokoreanin B (**39**) (Li et al., 1994), epimedonin E (**40**), F (**43**) (Nakashima et al., 2016), 4′-*O*-methyl limonianin (**41**), and limonianin (**42**) (Bacher et al., 2010).

### 3.2 The inhibitory effects of compounds on CML and CMA formation

**FIGURE 4:**
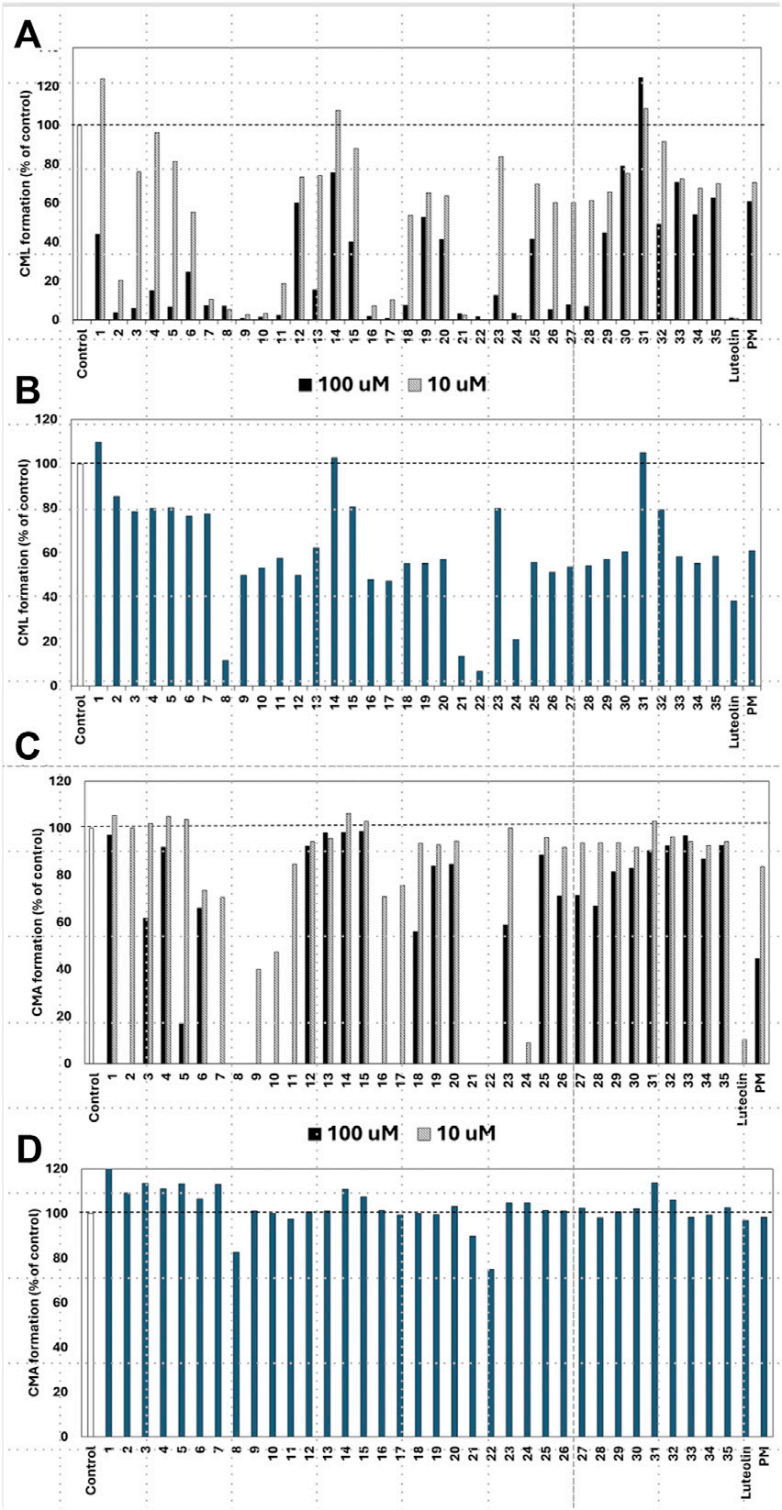
Effect of compounds from *Epimedii Herba* on CML formation. Gelatin (2.0 mg/mL) and ribose (30 mM) were incubated with the samples [**(A)**: 100 and 10 μM, **(B)** 1.0 μM] in 10 mM phosphate buffer at 37°C for 7 days. The CML content was determined using noncompetitive ELISA. Effect of compounds from *Epimedii Herba* on CMA formation. Gelatin (2.0 mg/mL) and ribose (30 mM) were incubated with samples [**(C)**: 100 and 10 μM, **(D)** 1.0 μM] in 100 mM sodium phosphate buffer at 37°C for 7 days. The CMA content was determined using noncompetitive ELISA (mean ± SD, n = 3).

Of the 43 compounds isolated and determined from EH, 1 mM DMSO solutions were prepared for 35 compounds, which were evaluated for their inhibitory activity against CML and CMA formation (Table 2). The results showed that samples **7**, **8**, **9**, **10**, **16**, **17**, **21**, **22**, and **24** had vigorous CML production inhibitory activity of more than 80% at 10 μM (Figure 4A). All the nine prenylflavonoids that exhibited significant inhibitory activity were aglycones. For further comparison, when the sample concentration was examined at 1.0 μM, samples **8**, **21**, **22**, and **24** showed more potent inhibition than pyridoxamine (PM) and luteolin as the positive controls (Figure 4B). Inhibitory formation of CMA also showed significant activity in the same compounds at 10 μM (Figure 4C). In contrast, weak inhibitory activity was observed for samples **8**, **21**, and **22**, and the other compounds displayed no activity at 1.0 μM (Figure 4D).

**TABLE 2:** Prenylflavonoids from *Epimedii Herba* used for inhibiting activity test against CML and CMA formation.

Sample no.	Compound name (no.)	Sample no.	Compound name (no.)
**1**	Icariin (**18**)	**19**	Koreanoside F (**16**)
**2**	Icariside I (**30**)	**20**	*Koreanoside L (**1**)
**3**	Icariside II (**23**)	**21**	Epimedonin E (**40**)
**4**	Icarisoside A (**24**)	**22**	Epicornunin B (**32**)
**5**	Epimedoside C (**5**)	**23**	Epimedokoreanin C (**15**)
**6**	Limonianin (**42**)	**24**	Epicornunin F (**33**)
**7**	8, 5′-diprenylapigenin (**37**)	**25**	Epimedin C (**9**)
**8**	Epimedokoreanin B (**39**)	**26**	Korepimeoside A (**25**)
**9**	8-prenyl luteolin (**31**)	**27**	Korepimeoside B (**26**)
**10**	Broussonol D (**38**)	**28**	Epimedigrandioside A (**27**)
**11**	Euchrestaflavanone A (**35**)	**29**	Epimedokoreanoside I (**14**)
**12**	Sagittatoside A (**19**)	**30**	Epimedin K (**11**)
**13**	Korepimedoside A (**28**)	**31**	Epimedin L (**12**)
**14**	*Koreanoside E1 (**2)**	**32**	Caohuoside B (**13**)
**15**	*Koreanoside E2 (**3**)	**33**	Epimedin I (**10**)
**16**	Epimedonin C (**36**)	**34**	Epimedin A (**7**)
**17**	Epimedonin F (**43**)	**35**	Epimedin B (**8**)
**18**	Koreanoside G (**17**)	* New compound	

When the compounds evaluated for activity were divided into glycosides and aglycones, the CML and CMA production inhibitory activities of the aglycons tended to be more pronounced. Compounds with vigorous CML inhibitory activity also showed intense CMA inhibitory activity, especially aglycones of the flavanone skeleton without an oxygen functional group at the three-position. However, among the glycosides, icariside I (**2**) and epimedoside C (**5**), in which glucose is attached only at the seven-position of the flavonol backbone, were active (Figures 4A, C). In contrast, no activity was observed for limonianin (**6**) or epimedokoreanin C (EK-C, **23**), even as aglycones. The γ,γ-dimethylallyl group was on the dimethylpyrane ring of **6**. Comparing **6** with epimedonin C (**16**), **16** showed activity even when a dimethylpyran ring was present on the B ring. In contrast, **16** and **23**, which share the same A- and C-ring moieties, showed a marked decrease in activity when the B ring became a cyclopentane ring. Furthermore, when comparing the activity at lower concentrations, epimedokoreanin B (**8**), epimedonin E (**21**), epicornunin B (**22**), and F (**24**) (Figure 4B) showed greater muscular CML inhibitory activity at 1 μM than PM and luteolin, which were used as control drugs. These four compounds also showed vigorous CMA production inhibitory activity of more than 80% at 10 μM (Figure 4C). Based on these results, the standard chemical structure of prenylflavonoids with both CML and CMA formation inhibitory activity is an aglycon of the luteolin-type flavanone skeleton, with prenyl groups at the eight-position of the A ring and the 5′position of the B ring and a catechol group on the B ring.

**FIGURE 5:**
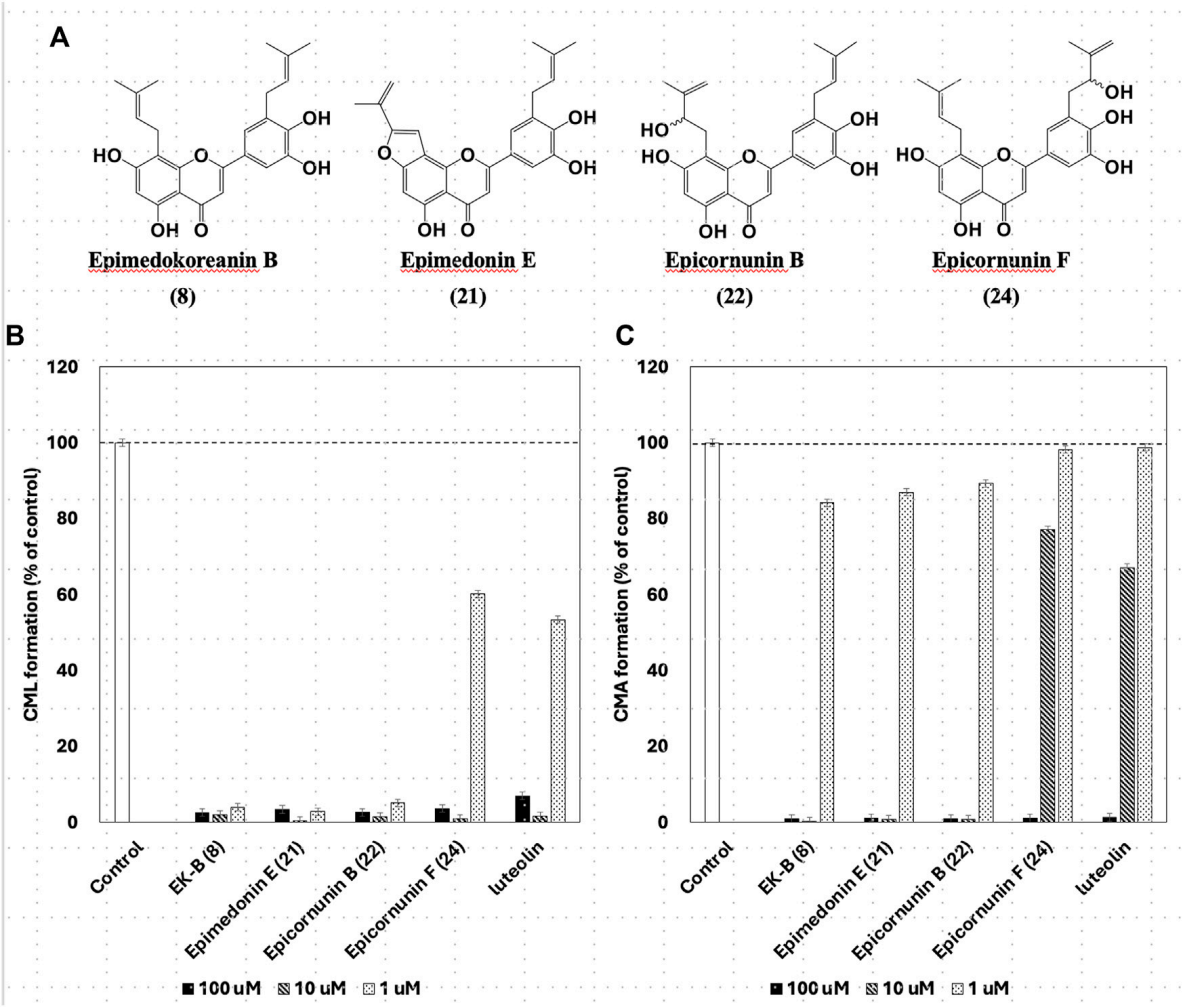
**(A)** The structures have strong inhibition activity of CML and CML formation. Effect of compounds from *Epimedii Herba* on CML **(B)** and CMA **(C)** formation. Type I collagen (1.5 mg/mL) and ribose (30 mM) were incubated with the compounds (100, 10, and 1 μM) in **(B)** 10 mM phosphate buffer; in **(C)** 100 mM sodium phosphate buffer at 37°C for 7 days. The CML and CMA content was determined using noncompetitive ELISA (mean ± SD, *n* = 3).

Instead of gelatin, type I collagen, which is present in the dermis, has been used to inhibit CML and CMA production. Four compounds, EK-B (**8**), epimedonin E (**21**), epicornunin B (**22**), and F (**24**) that showed significant inhibitory activity in the gelatin evaluation system, were used in this experiment. Prenylflavonoids (**8**, **21**, **22**, and **24**) in Figure 5A were added to a mixture of ribose (30 mM) and type I collagen (1.5 mg/mL), incubated at 37°C for 7 days, and then measured by ELISA using monoclonal anti-CML and anti-CMA antibodies. The amounts of CML and CMA generated in the type I collagen were determined by ELISA using monoclonal anti-CML and anti-CMA antibodies, respectively (Figure 5B; C). The results showed that the four prenylflavonoid compounds in EH had significant inhibitory activity against both CML and CMA. In particular, EK-B (**8**), epimedonin E (**21**), and epicornunin B (**22**) almost wholly inhibited CML formation even at concentrations of 1 μM (Figure 5B). These compounds also inhibit CMA entirely at a concentration of 10 μM (Figure 5C). In contrast, epicornunin F (**24**) showed inhibitory activity against CML and CMA formation, comparable to that of luteolin, which was used as a control drug.

Gelatin is a hydrolyzed and solubilized form of collagen that has the same amino acid sequence as collagen but a different steric structure. The CML and CMA inhibitory activities of the four prenylflavonoids (Figure 5A) were also observed in collagen, which has a different conformation. In other words, gelatin used as a mediocre protein reproduced the same inhibitory activity as expensive collagen. Thus, the usefulness of gelatin in screening prenylflavonoids for inhibition of CML and CMA production was confirmed.

### 3.3 Structure–activity relationships on CML and CMA formations

**FIGURE 6:**
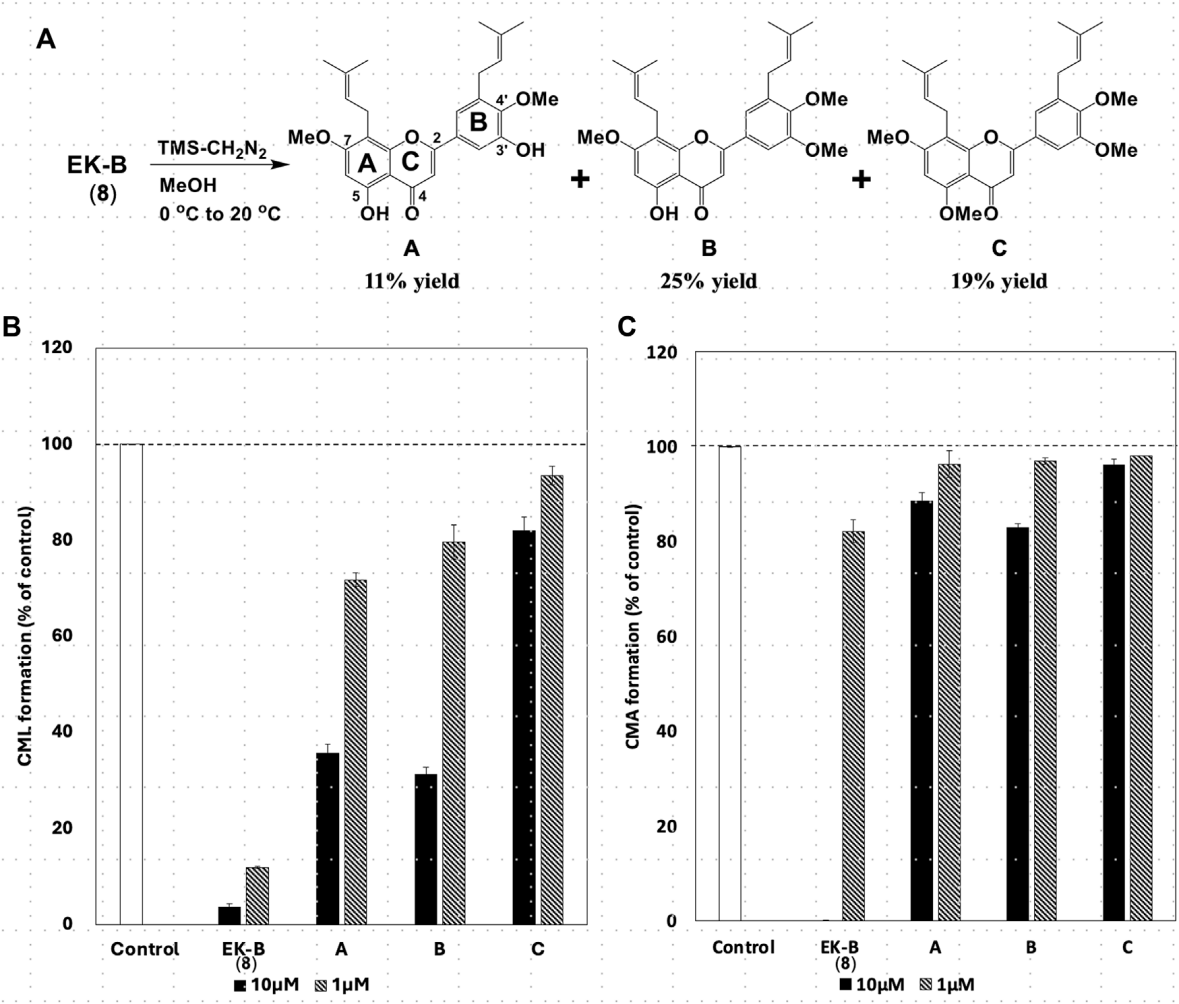
**(A)** Partial methylation of EK-B (**8**). Effect of methylated compounds **(A–C)** derived from EK-B (**8**) on **(B)** CML and **(C)** CMA formation. Gelatin (2.0 mg/mL) and ribose (30 mM) were incubated with the compounds (10 and 1 μM) in **(B)** 10 mM phosphate buffer; in **(C)** 100 mM sodium phosphate buffer at 37°C for 7 days. The CML and CMA content was determined using noncompetitive ELISA. Data are presented as the mean ± SD.

**FIGURE 7:**
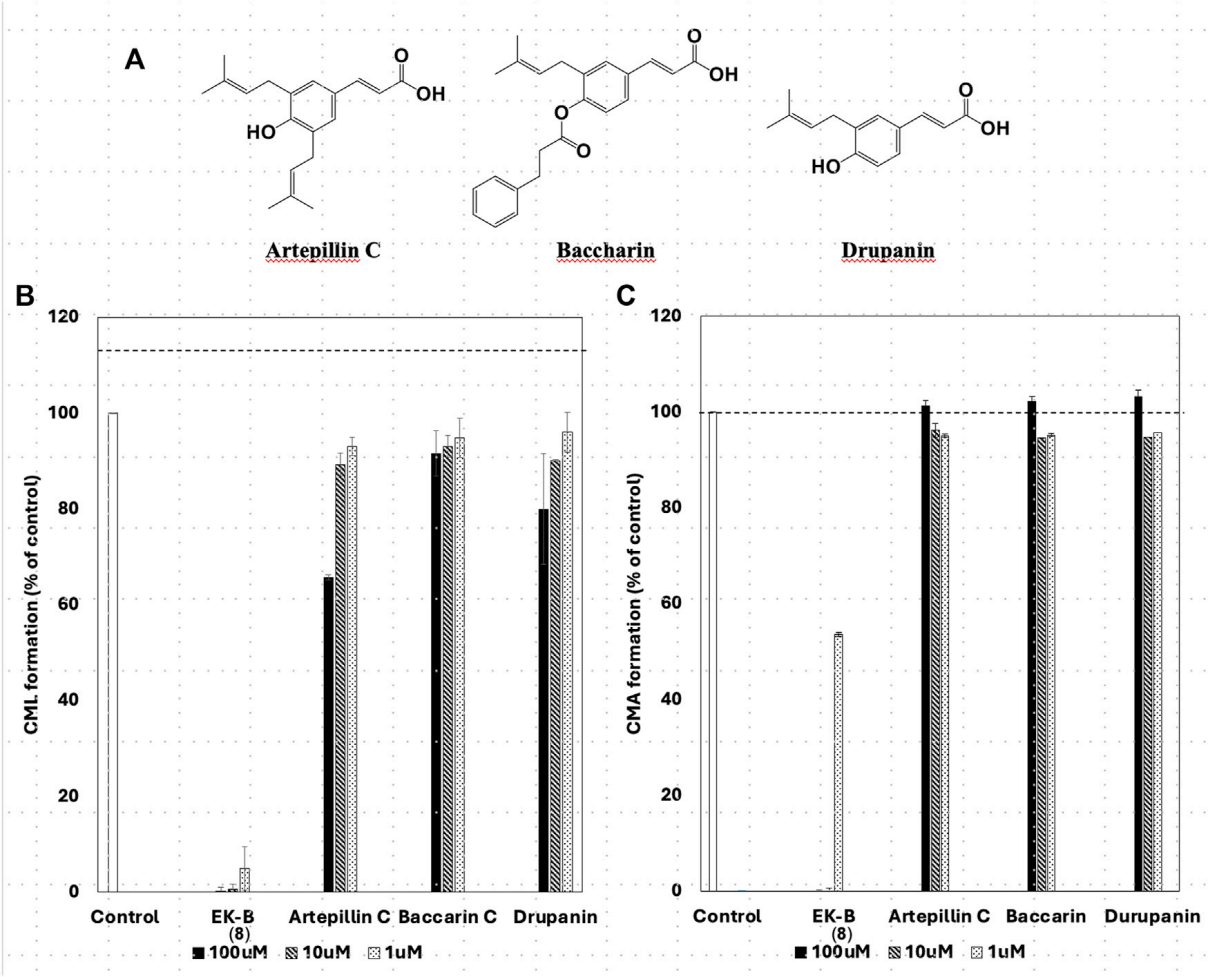
Chemical structures of prenylated cinnamic acid derivatives (PCAs) **(A)** artepillin C, baccharin, and drupanin. Comparative study of the effect of EK-B (**8**) and PCAs on **(B)** CML and **(C)** CMA formation. Gelatin (2.0 mg/mL) and ribose (30 mM) were incubated with the compounds (100, 10, and 1 μM) in **(B)** 10 mM phosphate buffer; in **(C)** 100 mM sodium phosphate buffer at 37°C for 7 days. The CML and CMA content was determined using noncompetitive ELISA. Data are presented as the mean ± SD.

All compounds with significant inhibitory activity against CML and CMA production (Figure 5A) contained a catechol group. To investigate the importance of the catechol group, we methylated the hydroxyl group of **8**, which had the most potent inhibitory activity and the highest yield. We then measured the inhibitory effects on CML and CMA formation. First, the four phenol hydroxyl groups of **8** were partially methylated using TMS-diazomethane. The reaction was monitored using silica gel TLC and stopped when the fully methylated form (**C**) was formed (Figure 6A). The resulting mixture containing the partially methylated product was separated and purified using silica gel to isolate the three products, along with raw material recovery. The chemical structures of the partially methylated forms **A**, **B**, and the fully methylated form **C** were determined from various NMR data. The yield of each compound was 11% for **A**, 25% for **B**, and 19% for **C**. The derivatives obtained (**A**–**C**) were tested for their CML and CMA formation inhibitory activities. All the methylated compounds showed lower CML and CMA formation inhibitory activities than **8** (Figures 6B, C). Compared to compounds **A**, **B**, and **C**, the CML formation inhibitory activity (Figure 6B, at 10 μM) was significantly reduced when the hydroxyl group attached to the C-5 in ring A was methylated as shown in compound **C**. Silica gel TLC (Hexane: acetone = 2:1 (*v/v*)) analysis of compound **B**, in which the hydroxyl group at position five of the B ring remains, and the fully methylated compound **C** showed that the *Rf* value of compound **B** was 0.75. In contrast, that of compound **C** was as low as 0.20. Furthermore, in HPLC analysis [column: YMC C_18_ (ϕ10 × 250 mm); flow rate: 2.0 mL/min; temperature: 40 °C; detection: RI], the retention time of compound **C** was 14.5 min, which was shorter than that of compound **B** (19.9 min). Thus, methylation of the hydroxyl group at the five-position of the A ring causes a marked change in the chemical and physical properties owing to the loss of the hydrogen bond with the carbonyl oxygen at the four-position of the C ring. Comparison of the CML and CMA inhibitory activities of **eight** reaffirmed the importance of the catechol group of the flavonoid B ring. They suggested that the hydrogen bond between the hydroxyl group at the five-position of the A ring and the carbonyl group at the four-position of the C ring is essential for the inhibitory activity against CML and CMA formation. Next, we focused on the prenyl group, a substructure other than the catechol group of **8**, which exhibits inhibitory activity. Therefore, we examined its contribution to the inhibitory activities of CML and CMA formation. In this study, three prenylated cinnamic acid derivatives, artepillin C, baccharin, and drupanin (Figure 7A), which are the main components of propolis (Rodrigues et al., 2020), were used as prenyl-related compounds for the inhibitory activity test. As shown in Figures 7B, C, the prenylated cinnamic acid derivatives used in this study did not inhibit CML or CMA formation. This suggests that neither prenylated cinnamic acid derivatives (two prenyl groups for artepillin C and one for drupanin) nor their aromatic esters (baccharin) were active; rather, the binding of prenyl groups to the flavonoid backbone, which contains phenolic hydroxyl groups, was responsible for the inhibition of CML and CMA formation. This suggests that the binding of a prenyl group to the flavonoid skeleton may enhance the inhibitory activity against CML and CMA formation.

In summary, the structure–activity relationship of the prenyl-related compounds from EH revealed that a catechol group in the B ring and prenyl groups at the eight and 5′positions are essential for the inhibitory activity against CML and CMA formation, that the hydroxyl group at the five-position is hydrogen bonded, and that the three-position does not have an oxygen functional group. In other words, compound **8**, which had the highest yield (0.084%) and the most potent inhibitory activity against CML and CMA formation, was found to be the active compound in the extract. Our findings are still experimental results at the test-tube level. However, we were able to clarify the partial structure of the prenylflavonoids required for anti-glycation activity. Although further studies should be conducted in animal models of diseases related to diabetes, atherosclerosis, and osteoporosis, these results suggest that compound **8** could be used as a therapeutic compound because it inhibits AGE formation and prevents the development of diabetic complications, such as diabetic nephropathy, retinopathy, and neuropathy, and age-related diseases, such as Alzheimer’s disease.

## Data Availability

The raw data supporting the conclusion of this article will be made available by the authors, without undue reservation.

## Abbreviations

AGEs, Advanced glycation end products; EH, *Epimedii Herba*; HMBCs, Heteronuclear multiple bond correlations; HMQ, Heteronuclear multiple quantum coherence; HDL, High density lipoprotein; HRESIMS, High-resolution electrospray ionization mass spectrometry; MeOH, Methanol; CMA, *Nω*-(carboxymethyl) arginine; CML, *Nε*-(carboxymethyl)lysine.
