# Supplementary material for: Prenylflavonoids isolated from Epimedii Herba show inhibition activity against advanced glycation end-products
Source: Front Chem. 2024 May 31;12:1407934. doi: 10.3389/fchem.2024.1407934 (PMC11176478; doi:10.3389/fchem.2024.1407934)
Supplement: Supplementary file 1 [file DataSheet2.docx]

Supplementary Material

Prenylflavonoids isolated from *Epimedii Herba* show inhibition activity against advanced glycation end-products

**Keisuke Nakashima, Hiroyuki Miyashita, Hitoshi Yoshimitsu, Yukio Fujiwara, Ryoji Nagai, Tsuyoshi Ikeda***

*** Correspondence:** Tsuyoshi Ikeda: tikeda@ph.sojo-u.ac.jp

Part 2 (2/2)

The spectral data of compounds **2** and **3** (**Figures S14**~**S27**).


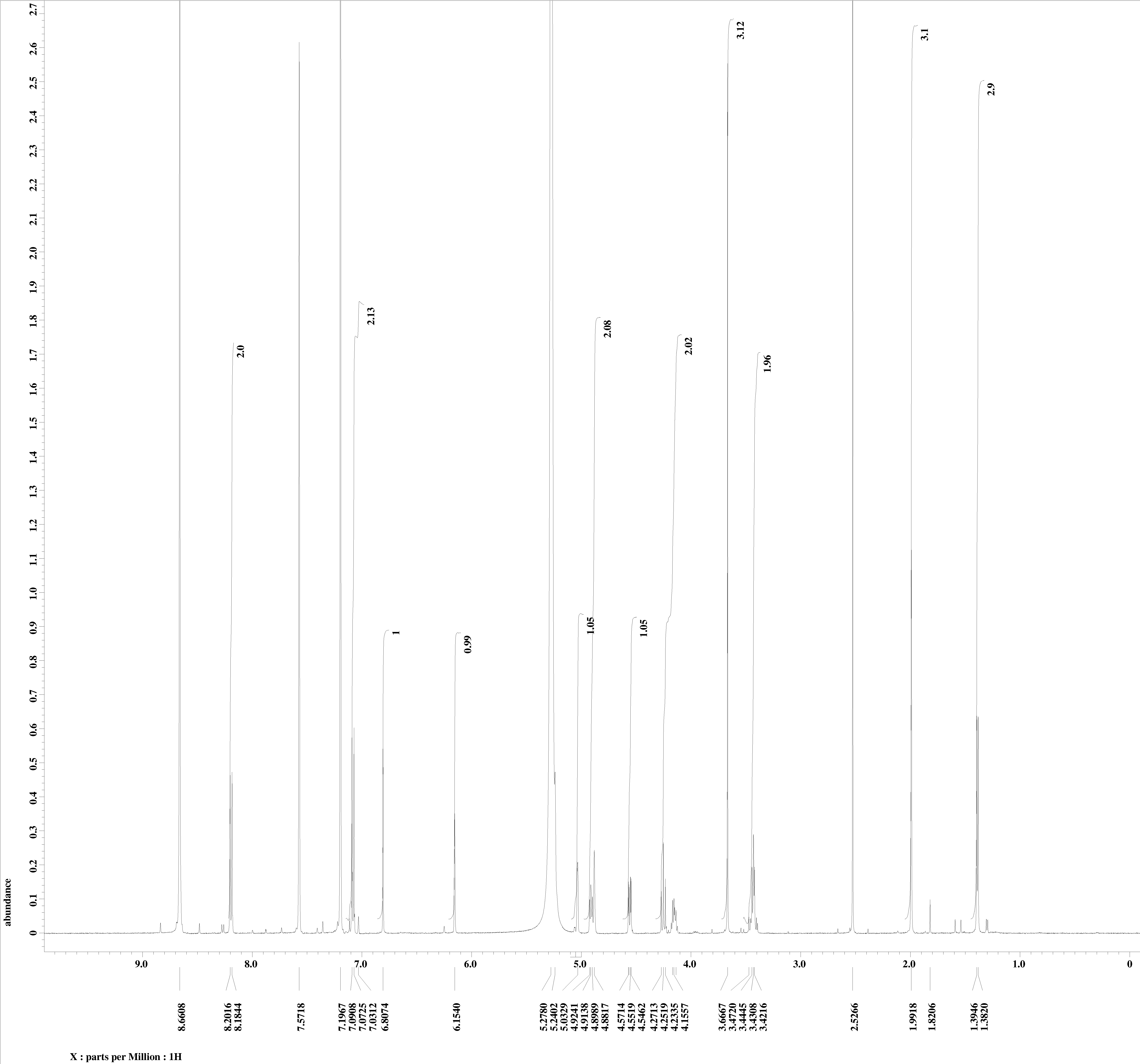


**Figure S14.** ^1^H NMR spectrum of **2** (in Pyridine-*d_5_*, 500 MHz)


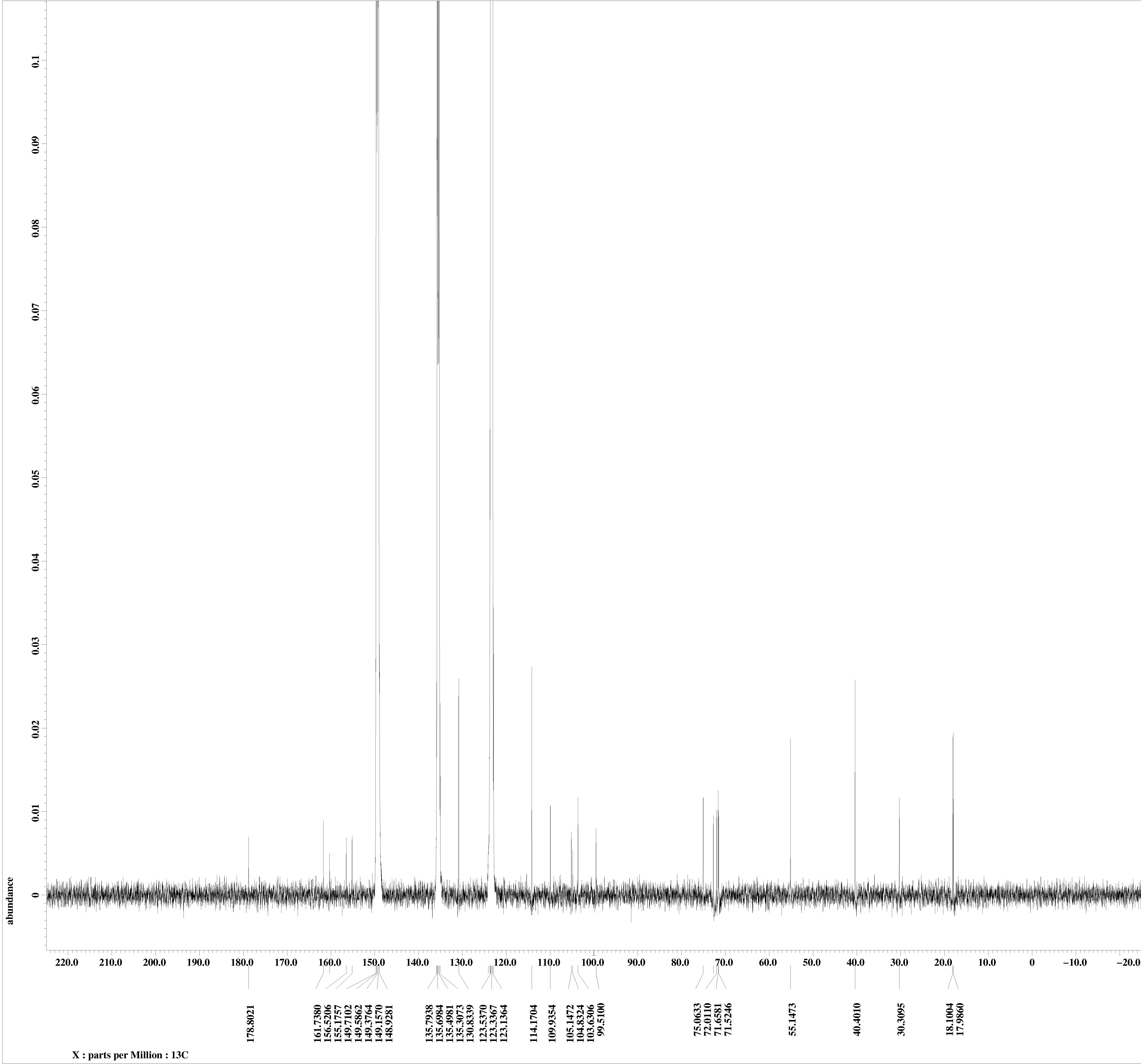


**Figure S15.** ^13^C NMR spectra of **2** (in Pyridine-*d_5_*, 125 MHz)


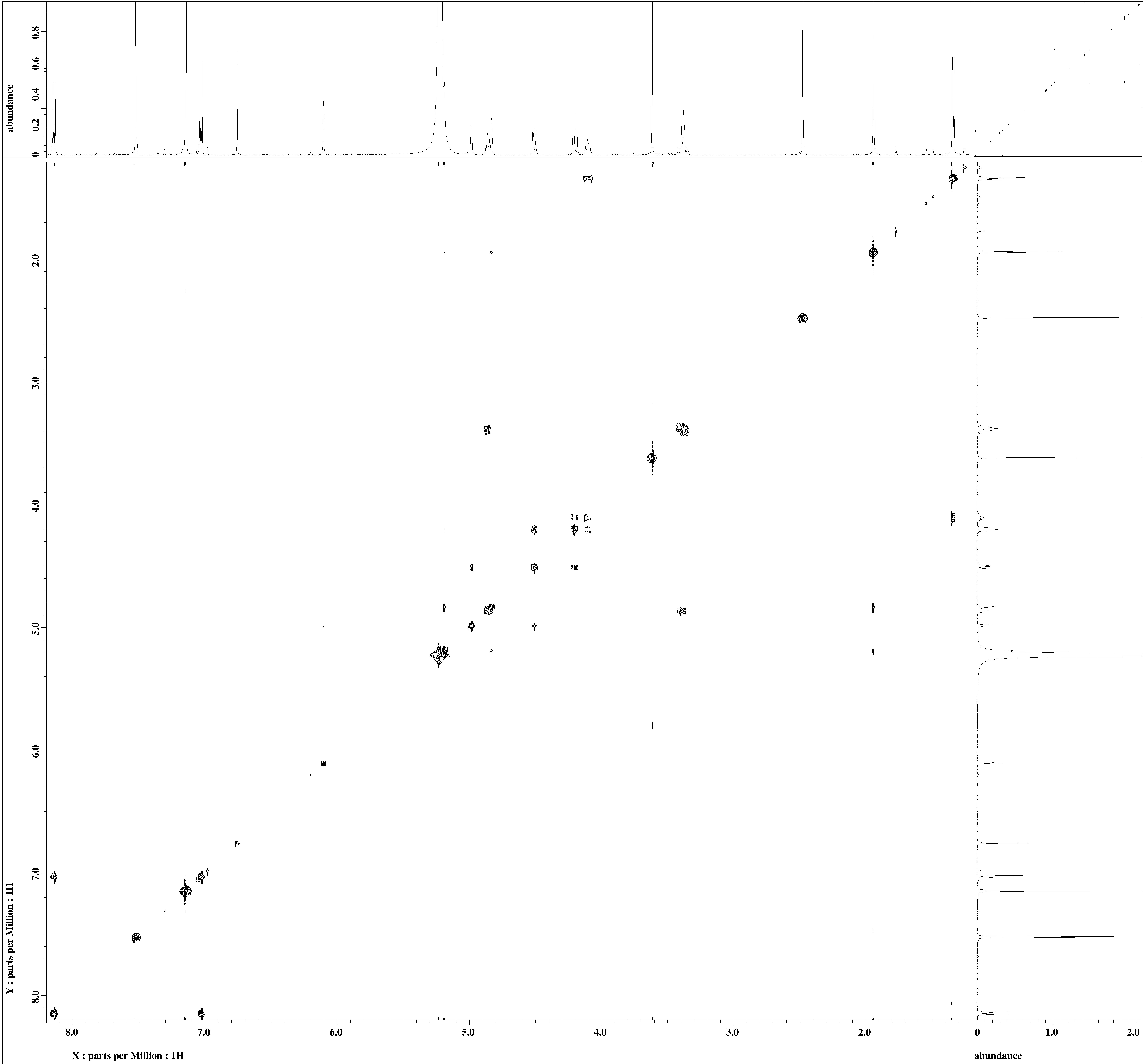


**Figure S16.** ^1^H-^1^H COSY spectrum of **2** (in Pyridine-*d_5_*, 500 MHz)


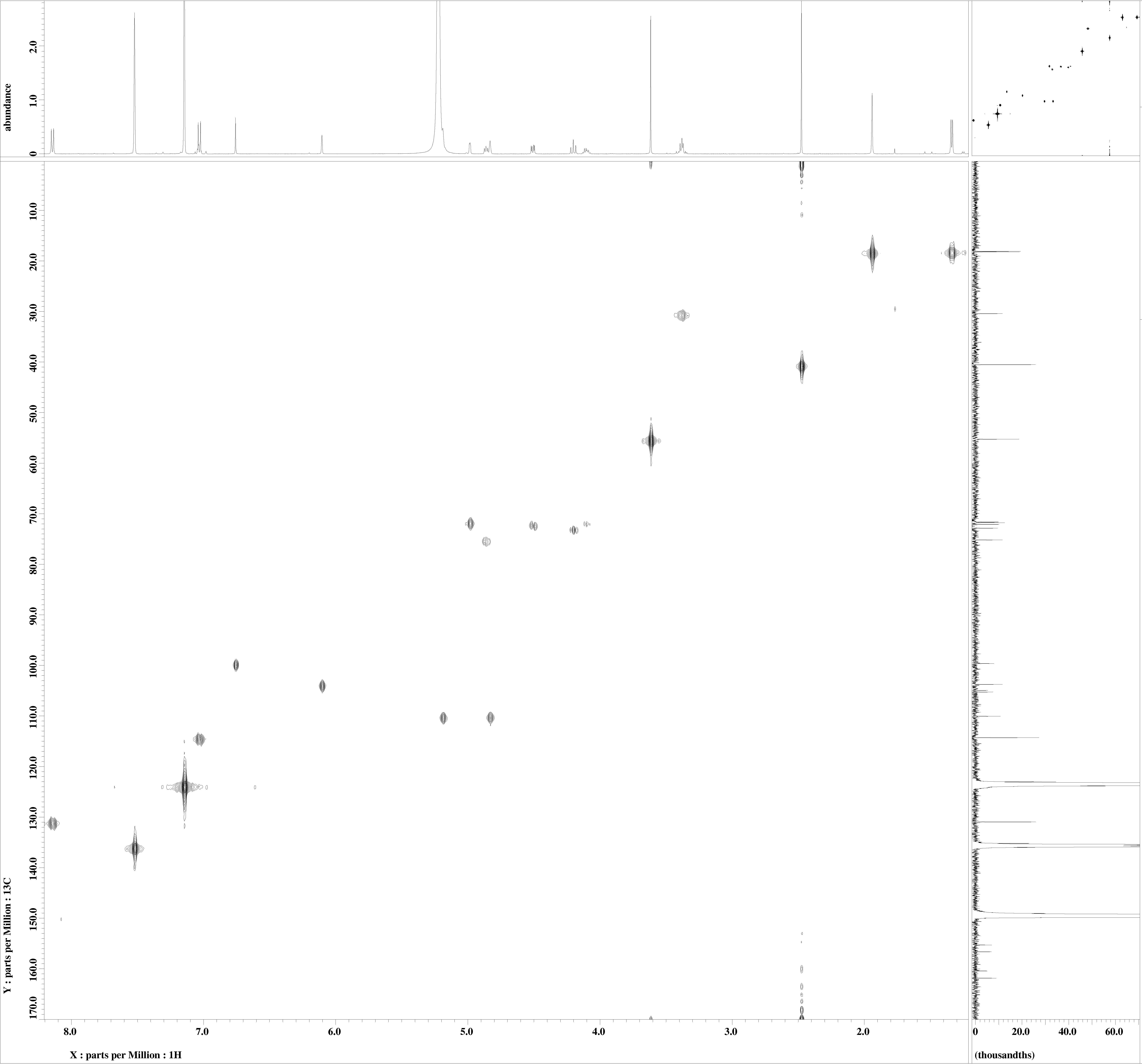


**Figure S17.** HMQC spectrum of **2** (in Pyridine-*d_5_*, 500 MHz)


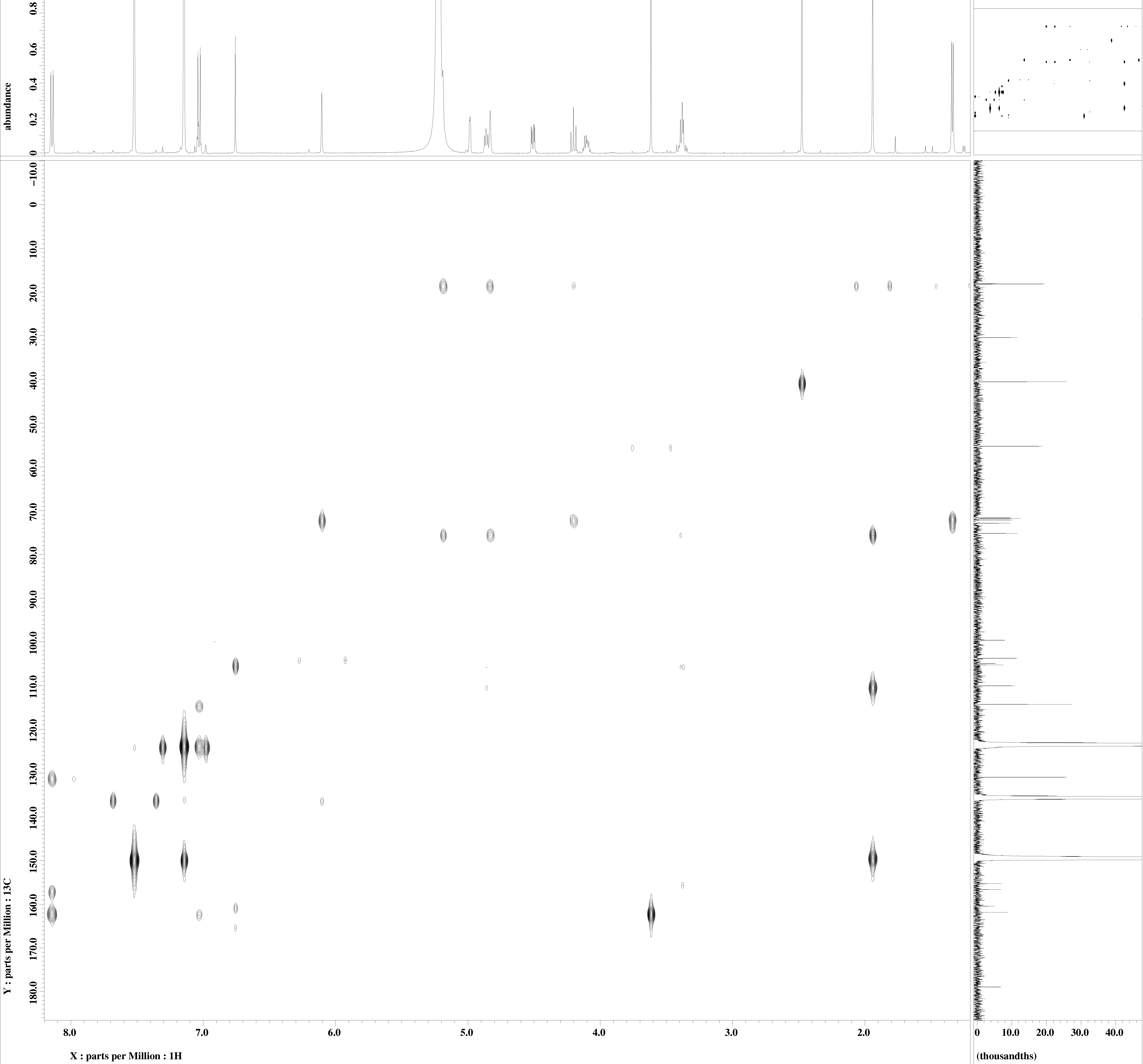


**Figure S18.** HMBC spectrum of **2** (in Pyridine-*d_5_*, 500 MHz)


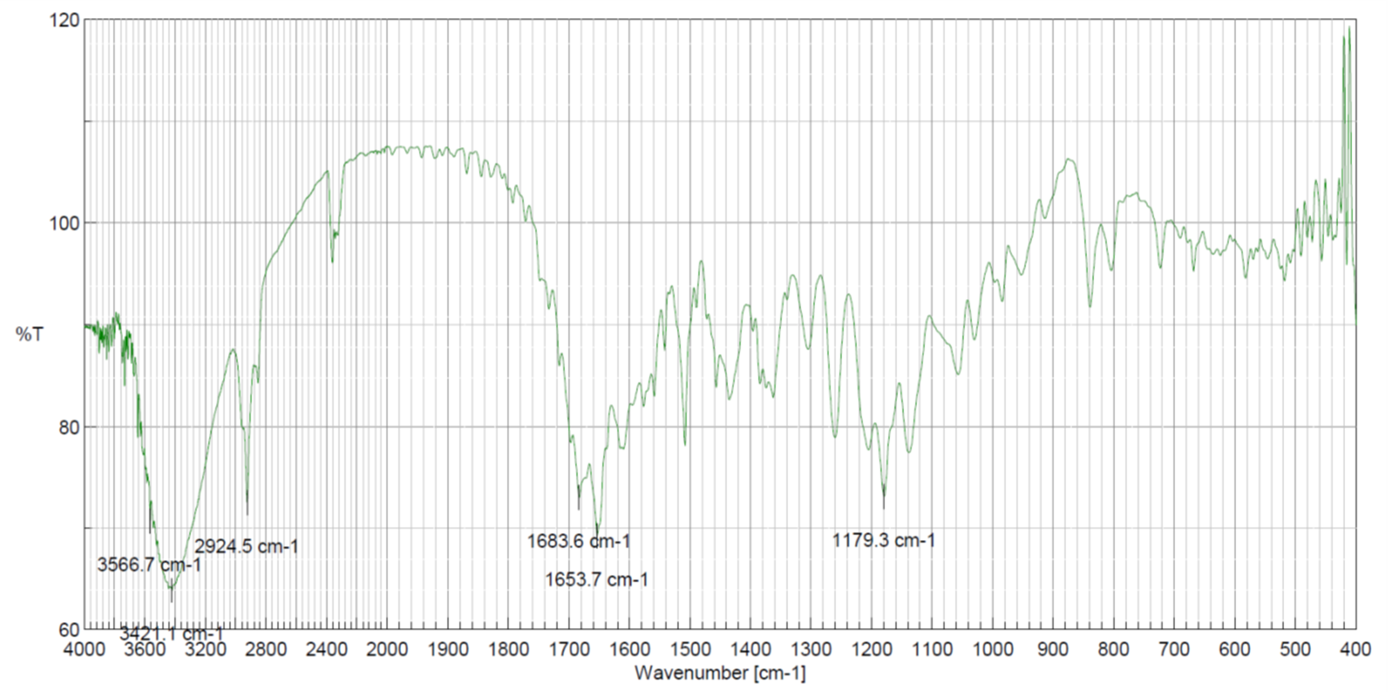


**Figure S19.** FT-IR spectrum of 2


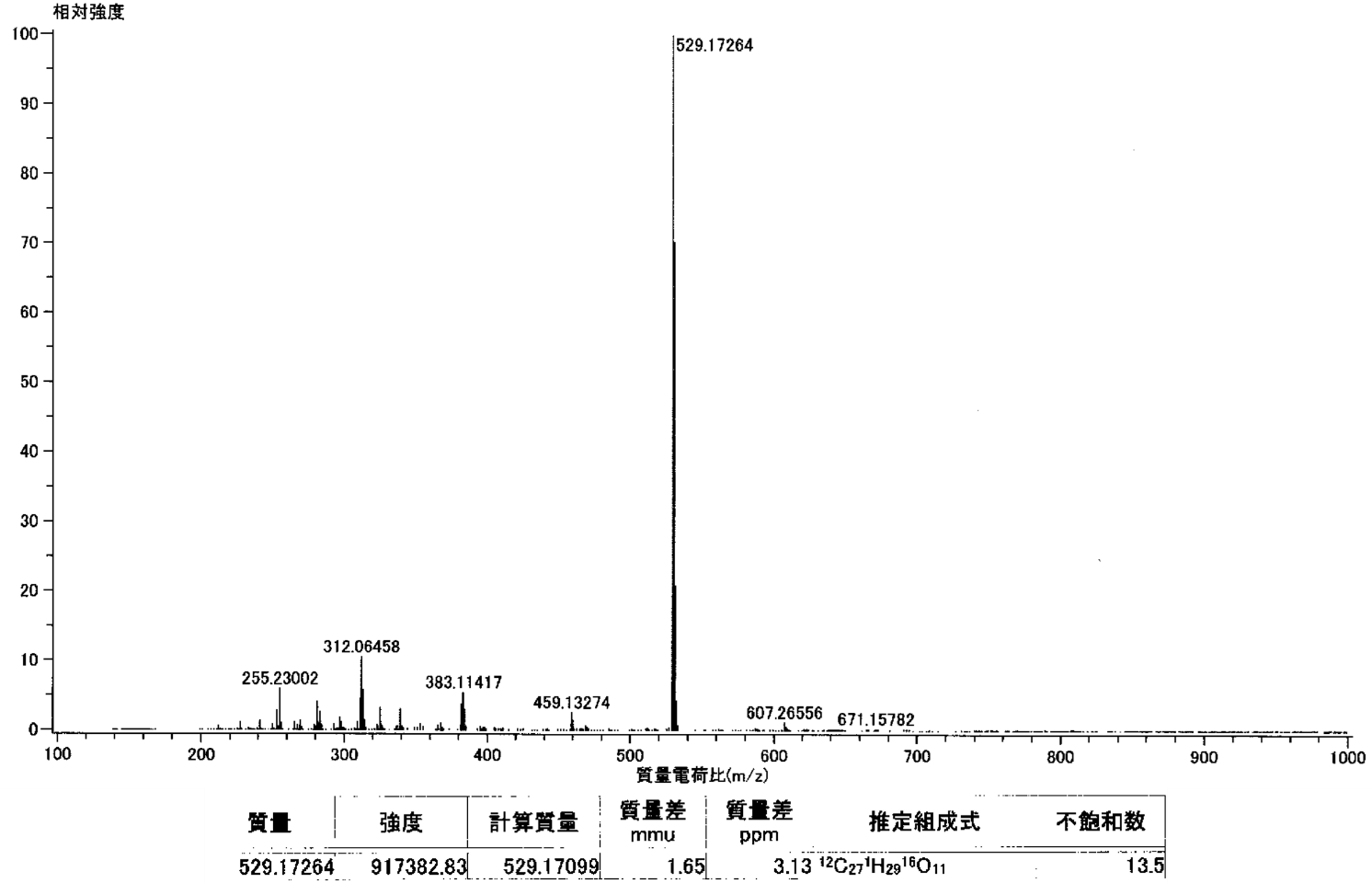


**Figure S20.** HR-Negative-ion ESI TOF-MS data of **2**


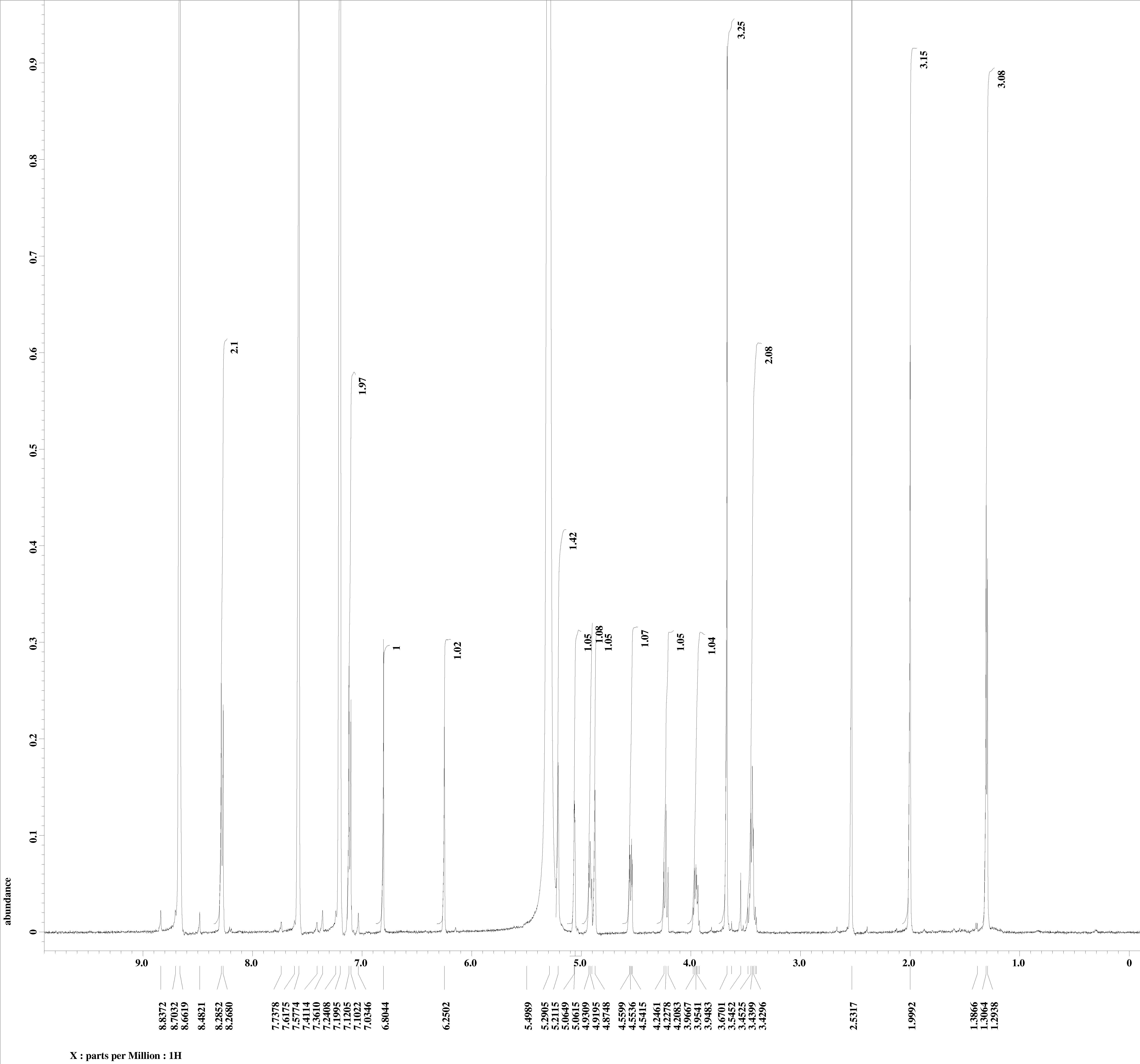


**Figure S21.** ^1^H NMR spectrum of **3** (in Pyridine-*d_5_*, 500 MHz)


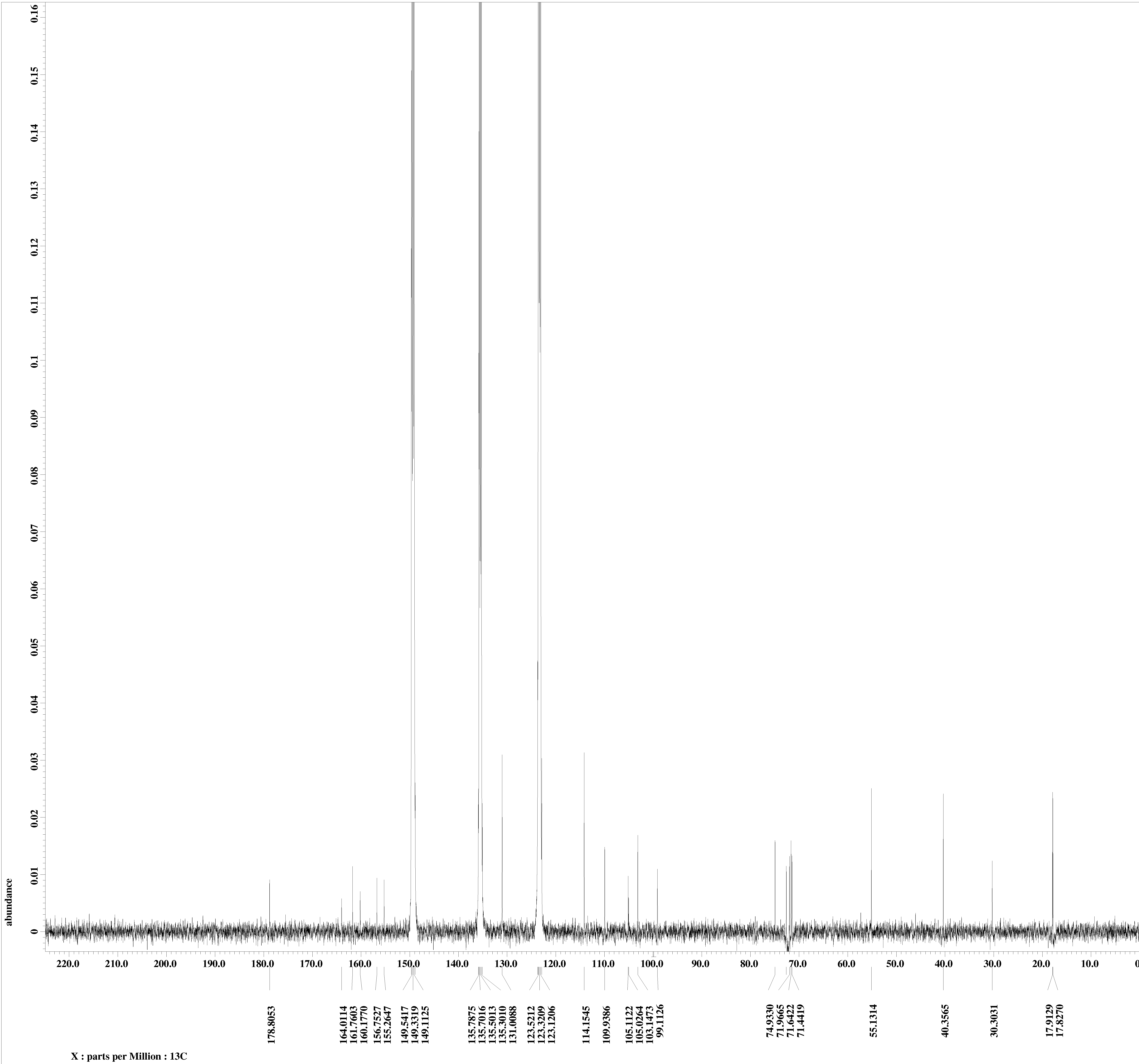


**Figure S22.** ^13^C NMR spectra of **3** (in Pyridine-*d_5_*, 125 MHz)


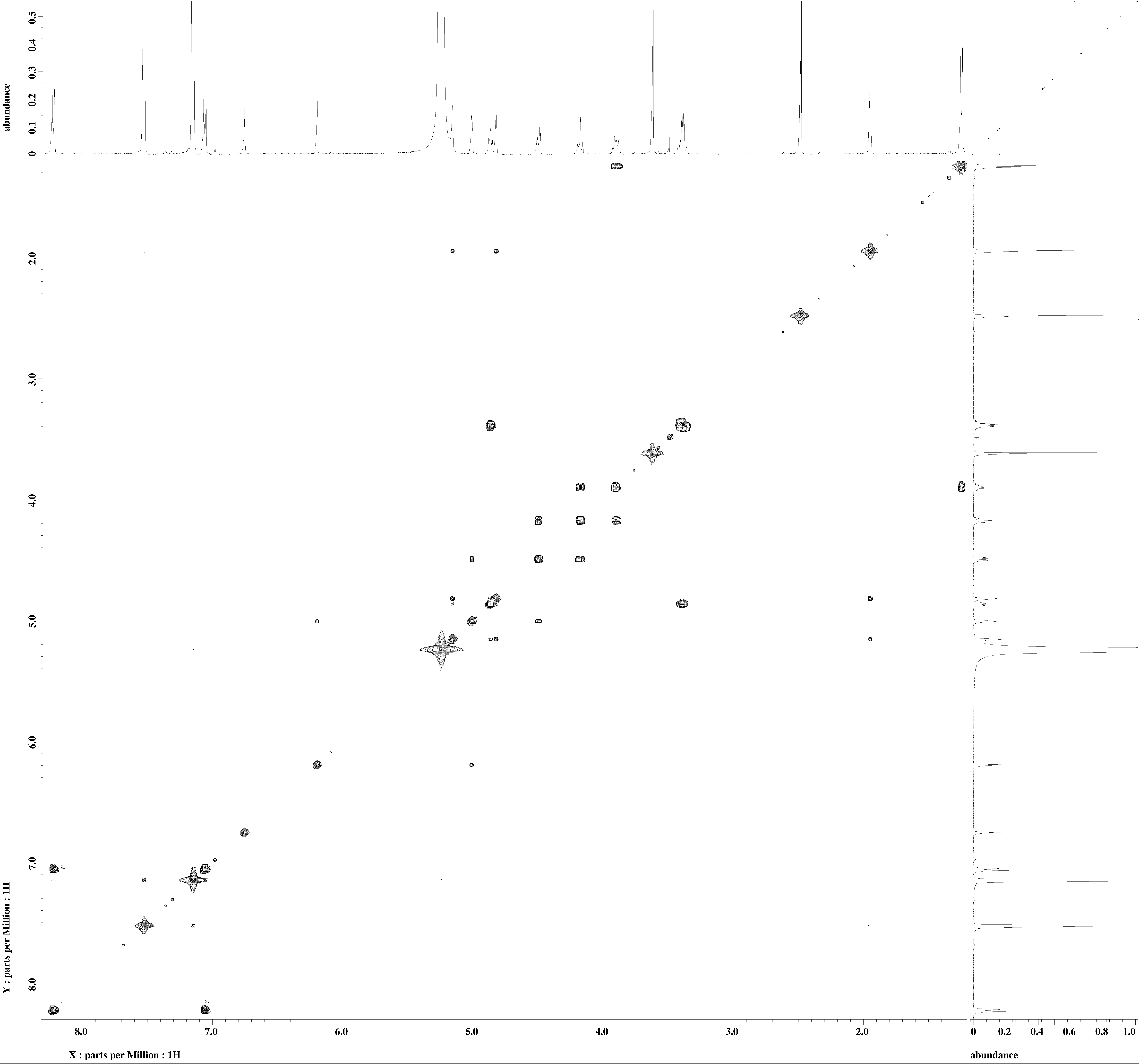


**Figure S23.** ^1^H-^1^H COSY spectrum of **3** (in Pyridine-*d_5_*, 500 MHz)


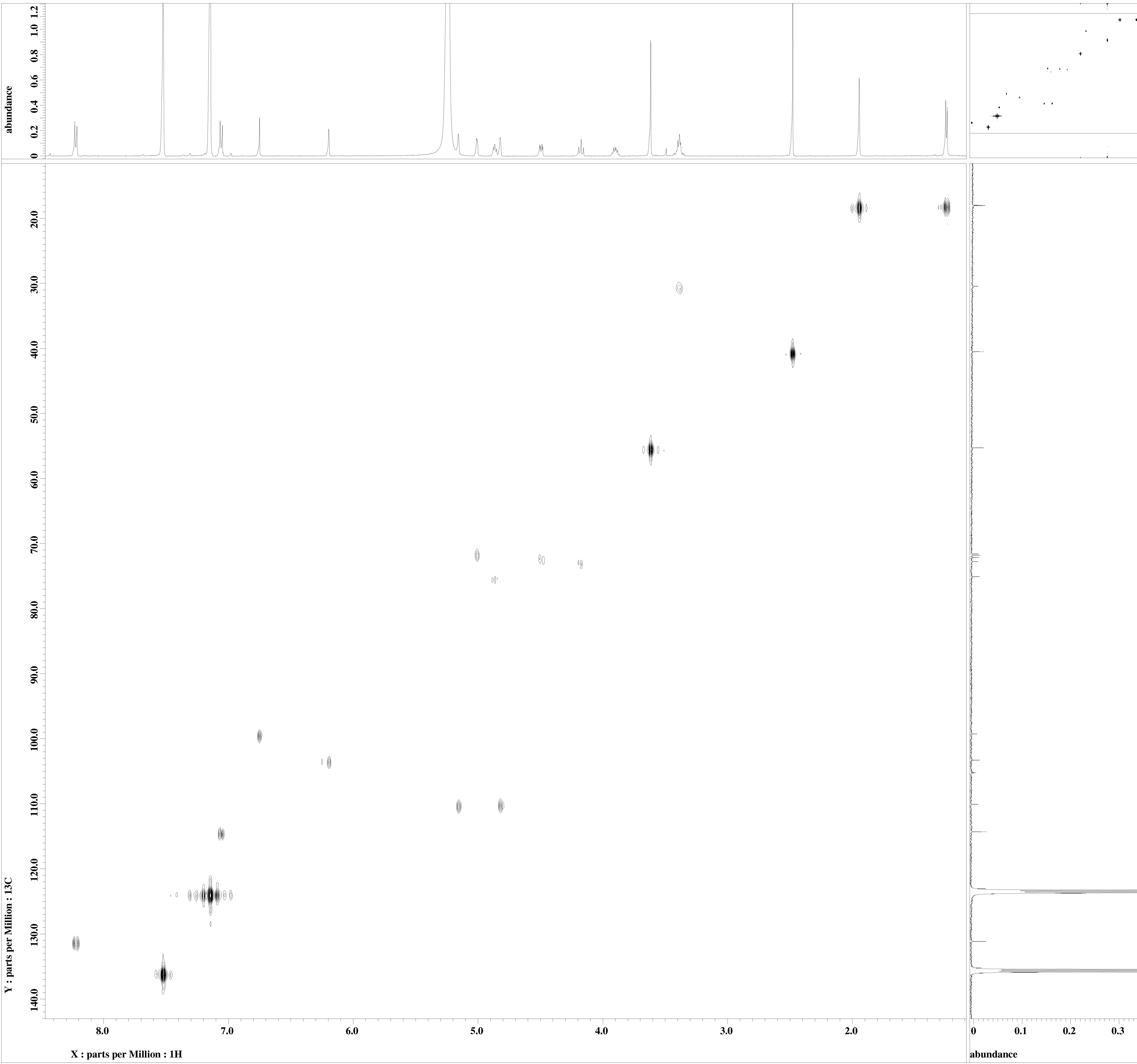


**Figure S24.** HMQC spectrum of **3** (in Pyridine-*d_5_*, 500 MHz)


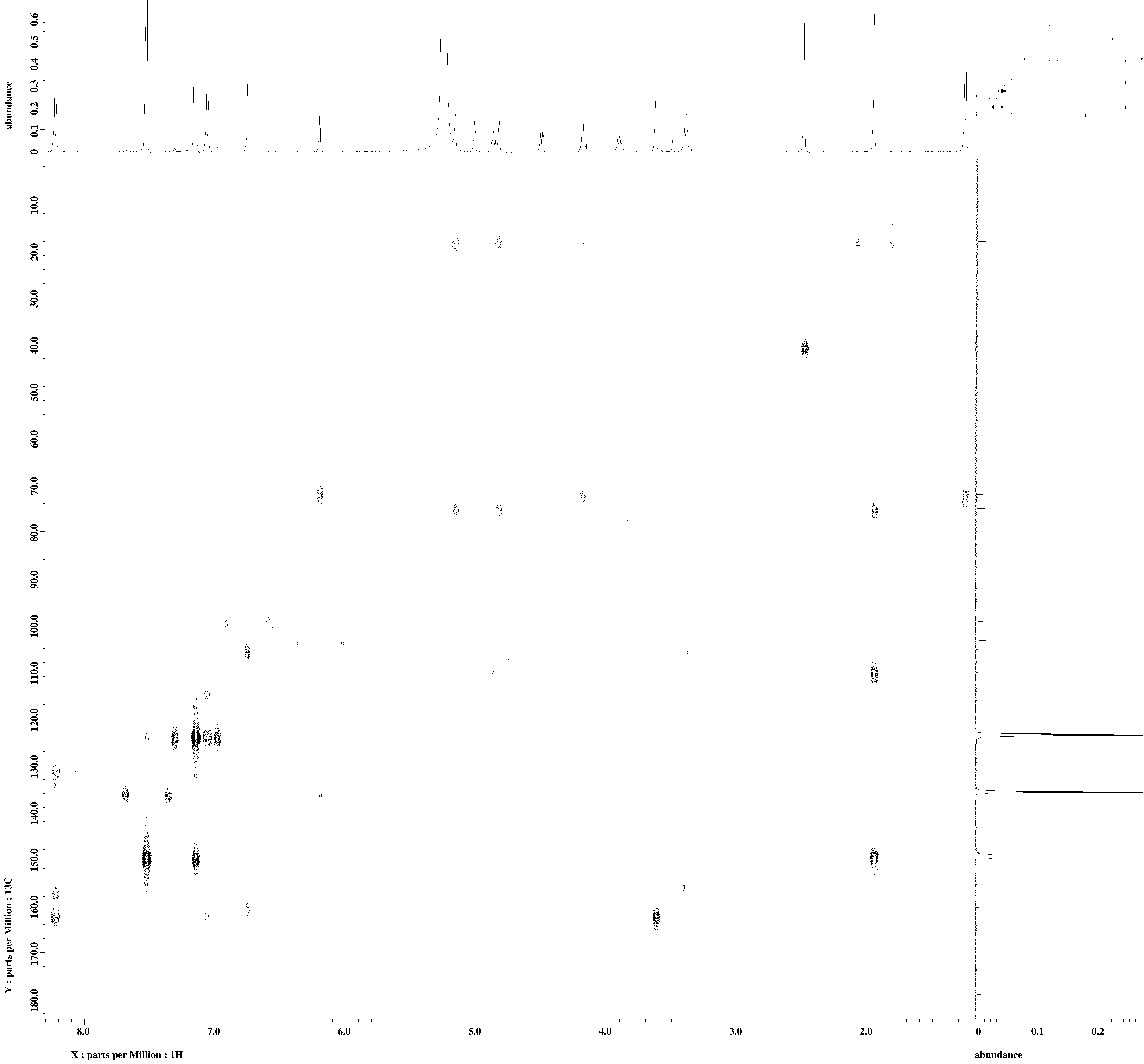


**Figure S25.** HMBC spectrum of **3** (in Pyridine-*d_5_*, 500 MHz)


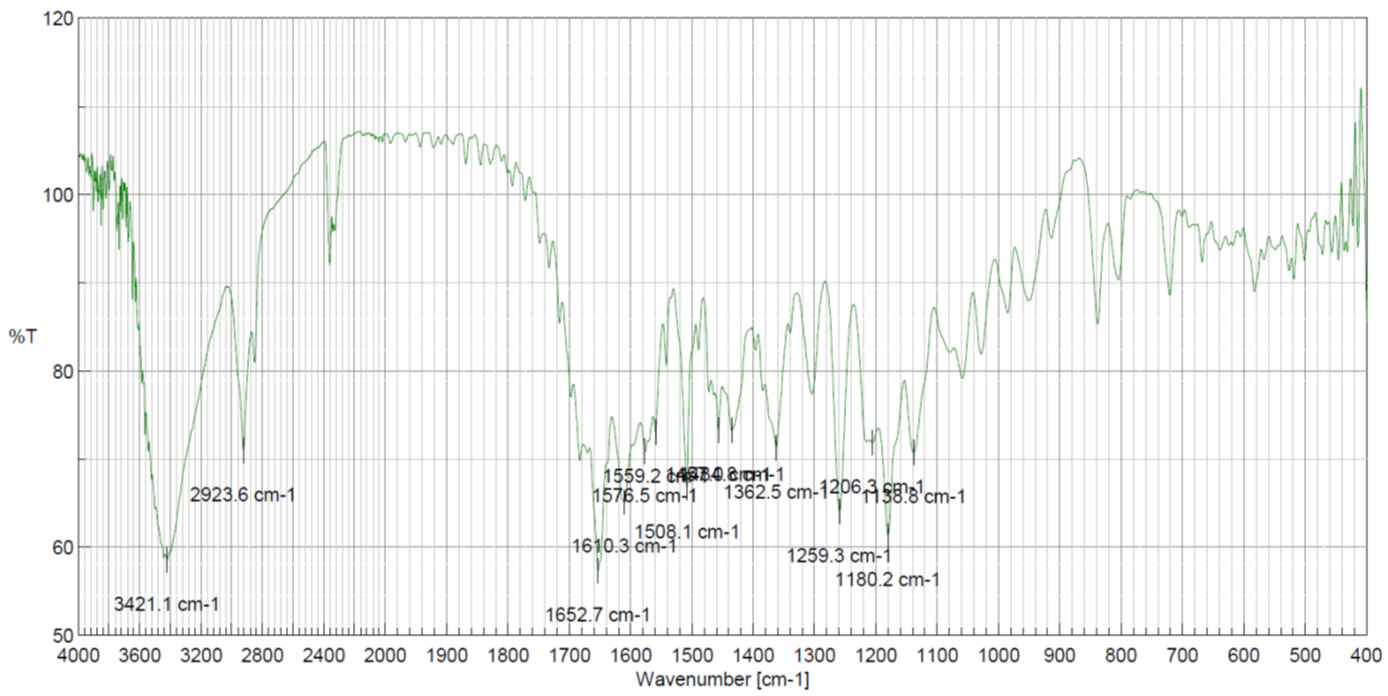


**Figure S26.** FT-IR spectrum of 3


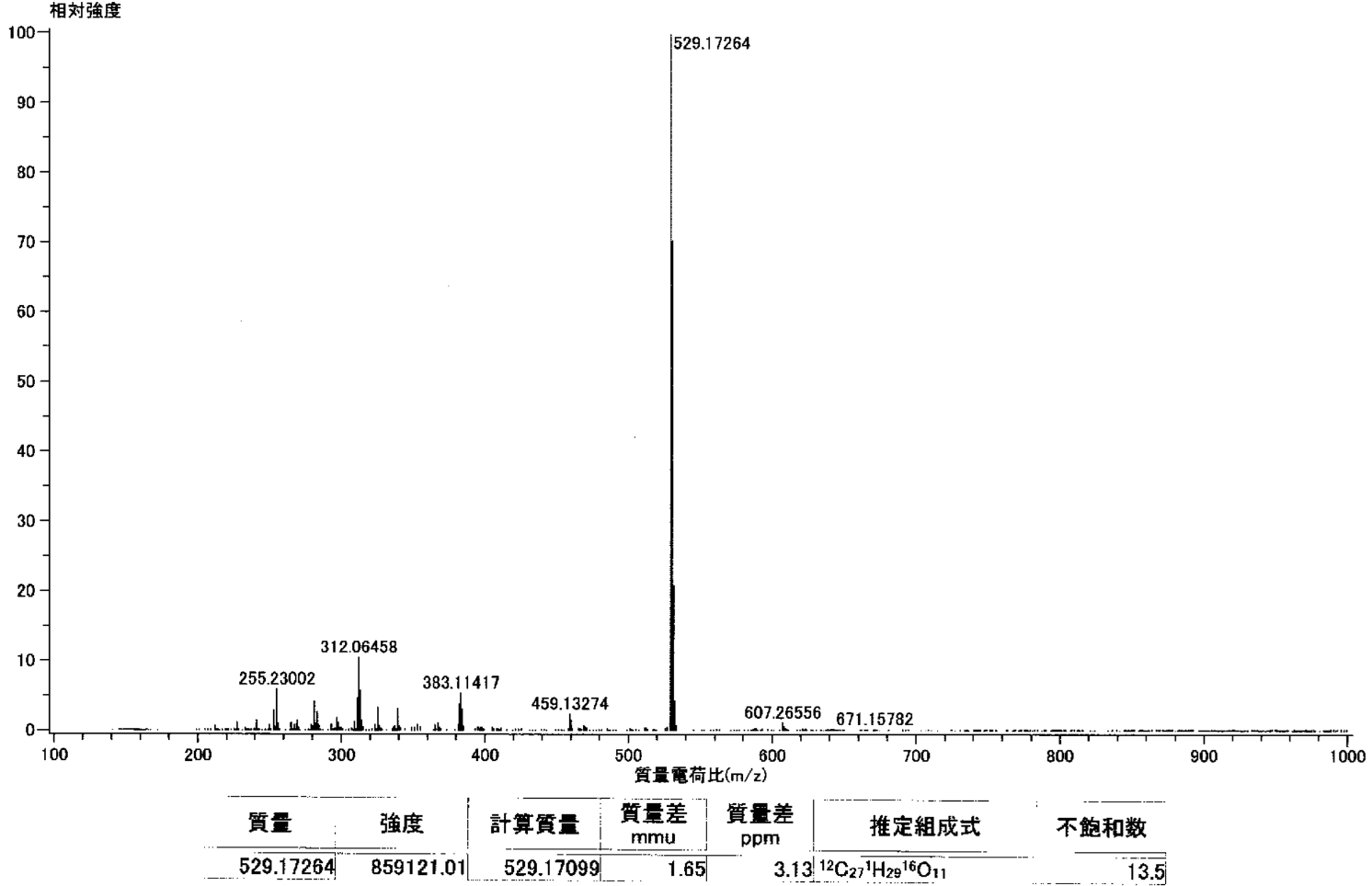


**Figure S27.** HR-negative-ion ESI TOF-MS data of **3**
