# Supplementary material for: Prenylflavonoids isolated from Epimedii Herba show inhibition activity against advanced glycation end-products
Source: Front Chem. 2024 May 31;12:1407934. doi: 10.3389/fchem.2024.1407934 (PMC11176478; doi:10.3389/fchem.2024.1407934)
Supplement: Supplementary file 2 [file DataSheet1.docx]

Supplementary Material

Prenylflavonoids isolated from *Epimedii Herba* show inhibition activity against advanced glycation end-products

**Keisuke Nakashima, Hiroyuki Miyashita, Hitoshi Yoshimitsu, Yukio Fujiwara, Ryoji Nagai, Tsuyoshi Ikeda***

*** Correspondence:** Tsuyoshi Ikeda: [tikeda@ph.sojo-u.ac.jp](mailto:tikeda@ph.sojo-u.ac.jp)

Part 1 (1/2)

# Supplementary Data

- 1. **Extraction and isolation procedure of compounds 1~43 from EH**.

EH (3.0 kg) was extracted twice with MeOH by sonication for 6 hr (30 min×12) at room temperature. The extract was concentrated under reduced pressure to afford a residue (485 g). The residue was partitioned between *n*-hexane and 80 % MeOH, after which the 80 % MeOH layer was concentrated to yield a residue (408 g), which was loaded onto an MCI-gel CHP20P column (φ50×300 mm) and eluted with an H_2_O–MeOH gradient (0, 50, and 100 % MeOH; 1.5 L of each gradient solution) to give 3 fractions (frs. 1~3). Fr. 2 (46.5 g, eluted by 50 % MeOH) was further applied to the MCI gel CHP20P column (φ40×300 mm) and eluted with an H_2_O–MeOH gradient (20, 30, 40, 50, 60, 70, 80, 90, and 100 % MeOH; 1.5 L of each gradient solution) to yield 8 fractions (frs. 2-1 to 2-8). Fr. 2-6 (5.3 g) was subjected to Sephadex LH-20 (φ20×1000 mm, using MeOH as mobile phase) to give 3 fractions (frs. 2-6-1 to 2-6-3). Fr. 2-6-3 (190 mg) was loaded onto μ-Bonda Pak C_18_ column chromatography (φ25×200 mm) and eluted with an H_2_O–MeOH gradient (60, 70, 80, and 90 % MeOH; 135 mL of each gradient solution) to yield 6 fractions (frs. 2-6-3-1 to 2-6-3-6). Fr. 2-6-3-5 (10.3 mg) and 6 (1.1 mg) was purified via preparative HPLC (Cosmosil 5C_18_ AR-II (φ10×250 mm, eluted with 70% MeOH, each) and isolated compounds **5** (4.5 mg) and **6** (0.5 mg), respectively.Fr. 2-7 (5.1 g) subjected to Sephadex LH-20 column (φ20×1000 mm, eluted with MeOH) to give 4 fractions (frs. 2-7-1 to 2-7-4). Fr. 2-7-2 (436 mg) was loaded onto μ-Bonda Pak C_18_ column chromatography (φ25×200 mm) and eluted with an H_2_O–MeOH gradient (60, 70, 80, and 90 % MeOH; 135 mL of each gradient solution) to afford 6 fractions (frs. 2-7-2-1 to 2-7-2-6). Fr. 2-7-2-2 (34.1 mg) was further applied to SiO_2_ (φ10×100 mm, CHCl_3_: MeOH: H_2_O = 8:2:0.2 (*v/v*)) after which it was purified via preparative HPLC [Cosmosil AR-II (φ10×250 mm, 70% MeOH)] to give compounds **7** (1.0 mg), **8** (0.5 mg), **9** (2.2 mg). Fr. 2-7-2-4 (145 mg) was loaded onto SiO_2_ (φ10×50 mm, CHCl_3_: MeOH: H_2_O = 8:2:0.2 (*v/v*)), after which it was purified via preparative HPLC [Cosmosil AR-II ODS (φ10×250 mm, eluted with 65% MeOH)] to give compounds **10** (6.1 mg), **11** (2.0 mg), **12** (6.2 mg), **13** (14.9 mg), and **14** (12.1 mg). Fr. 2-7-3 (370 mg) was loaded onto μ-Bonda Pak C_18_ column chromatography and eluted with an H_2_O–MeOH gradient (50, 60, 70, 80, 90 and 100 % MeOH), after which it was purified via SiO_2_ (CHCl_3_: MeOH: H_2_O = 8:2:0.2 (*v/v*)) and preparative HPLC (Cosmosil AR-II ODS (eluted with 70% MeOH) to afford compound **4** (1.8 mg).

Fr. 3 (65.0 g) was further applied to the MCI gel CHP20P column (φ40×300 mm) and eluted with an H_2_O–MeOH gradient (40, 50, 60, 70, 80, 90, and 100 % MeOH; 1.5 L of each gradient solution) to yield 8 fractions (frs. 3-1 to 3-8). Fr. 3-4 (9.0 g) was loaded on Sephadex LH-20 (φ20×1000 mm, eluted with MeOH) to give 5 fractions (frs. 3-4-1 to 3-4-5). Fr. 3-4-2 (286 mg) subjected to μ-Bonda Pak C_18_ column chromatography (φ25×200 mm) and eluted with an H_2_O–MeOH gradient (50, 60, 70, 80, 90 and 100 % MeOH; 135 mL of each gradient solution) and the fraction eluted with 60% MeOH was applied to preparative HPLC (X-Bridge Prep C_18_ (φ10×250 mm, eluted with 65% MeOH) to afford compound **15** (4.0 mg). A part of fr. 3-4-3 (500 mg) was loaded onto μ-Bonda Pak C_18_ column chromatography (φ25×200 mm) and eluted with an H_2_O–MeOH gradient (50, 60, 70, 80, 90% MeOH; 135 mL of each gradient solution) to give 6 fractions (frs. 3-4-3-1 to 3-4-3-6). Fr. 3-4-3-3 (41.4 mg) was subjected to SiO_2_ (φ10×130 mm, CHCl_3_: MeOH: H_2_O = 20:1:0~ 8:2:0.2 (*v/v*)) to yield 6 fractions (frs. 3-4-3-3-1 to 3-4-3-3-6). Fr. 3-4-3-3-2 (13.2 mg) was purified via two preparative HPLC columns [X-Bridge Prep. C_18_ (φ10× 50 mm, eluted with 65% MeOH) and YMC Triart PFP (φ4.6×250 mm, eluted with 60% MeOH)] to give compound **16** (2.3 mg). Fr. 3-4-3-3-3 (16.3 mg) was applied to preparative HPLC [Atlantis Prep. T3 C_18_ (φ10×250 mm, eluted with 70% MeOH)] to afford compounds **2** (5.1 mg) and **3** (5.5 mg). Fr. 3-4-3-4 (55.9 mg) was subjected to SiO_2_ (φ10×120 mm, CHCl_3_: MeOH: H_2_O = 9:1:0.1) (*v/v*)) and purified via preparative HPLC [X-Bridge Prep. C_18_ (φ10×250 mm, eluted with 60% MeOH)] to give compound **17** (4.1 mg). Fr. 3-4-3-5 (160 mg) was loaded onto SiO_2_ (φ10×100 mm, CHCl_3_: MeOH: H_2_O = 9:1:0.1 (*v/v*)) to yield 9 fractions (frs. 3-4-3-5-1 to 3-4-3-5-9). Fr. 3-4-3-5-7 (31.5 mg) purified via preparative HPLC [Sunfire Prep C_18_ (φ10×250 mm, eluted with 80% MeOH)] to afford compound **18** (28.9 mg). Fr. 3-4-3-5-9 (71.5 mg) was purified via preparative HPLC [Sunfire Prep C_18_ (φ10×250 mm, eluted with 70% MeOH)] to give compound **19** (47.3 mg) and **20** (9.8 mg). Fr. 3-4-4 (660 mg) was subjected to Sephadex LH-20 (φ20×1000 mm, eluted with MeOH) to yield 5 fractions (frs. 3-4-4-1 to 3-4-4-5). Fr. 3-4-4-2 (285.9 mg) was subjected to μ-Bonda Pak C_18_ column chromatography (φ25×200 mm) and eluted with an H_2_O–MeOH gradient (60, 70, 80, 90 % MeOH; 135 mL of each gradient solution) to give 9 fractions (frs. 3-4-4-2-1 to 3-4-4-2-9). Fr. 3-4-4-2-2 (55.6 mg) was purified via preparative HPLC (PFP (φ4.6×250 mm, eluted with 65% MeOH) to afford compound **21** (8.0 mg). Fr. 3-4-4-2-3 (77.1 mg) was subjected to SiO_2_ (φ15×90 mm, CHCl_3_: MeOH: H_2_O = 8:2:0.2 (*v/v*)) and purified HPLC [X-Bridge Prep C_18_ (φ10×250 mm, eluted with 70% MeOH)] to give compound **22** (6.0 mg). Fr. 3-4-4-2-5 (59.1 mg) was purified via preparative HPLC [X-Bridge Prep C_18_ (φ10×250 mm, eluted with 70% MeOH)] to give compound **23** (55.1 mg). Fr. 3-4-4-2-8 (3.0 mg) was also purified via preparative HPLC [X-Bridge Prep C_18_ (φ10×250 mm, eluted with 80% MeOH) to afford compound **1** (2.5 mg). Fr. 3-4-4-3 (184 mg) was loaded on μ-Bonda Pak C_18_ column chromatography (φ25×200 mm) and eluted with an H_2_O–MeOH gradient (50, 60, 70, 80, 90 % MeOH; 135 mL of each gradient solution) and purified via SiO_2_ (φ10×100 mm, CHCl_3_: MeOH: H_2_O = 8:2:0.2 (*v/v*)) to give compound **24** (27.2 mg). Fr. 3-5 (10.4 g) was loaded on Sephadex LH-20 (φ20×1000 mm, eluted with MeOH) to yield 5 fractions (frs. 3-5-1~ 3-5-5). Fr. 3-5-3 (1.23 g) was subjected to μ-Bonda Pak C_18_ column chromatography (φ25× 200 mm) and eluted with an H_2_O–MeOH gradient (60, 70, 80, 90 % MeOH; 135 mL of each gradient solution) to afford 5 fractions (frs. 3-5-3-1~ 3-5-3-5). Fr. 3-5-3-4 (206 mg) was loaded onto SiO_2_ (φ20×120 mm, CHCl_3_: MeOH: H_2_O = 9:1:0.1; (*v/v*)) to yield 8 fractions (frs. 3-5-3-4-1 to 3-5-3-4-8). Fr. 3-5-3-4-4 (11.5 mg) was purified via preparative HPLC [Cosmosil AR-II C_18_ (φ10× 250 mm, eluted with 70% MeOH)] to give compound **25** (5.9 mg) and **26** (4.5 mg). Fr. 3-5-3-4-5 (19.7 mg) was purified via two preparative HPLC columns [Cosmosil π Nap (φ10× 250 mm, eluted with 60% MeOH) and Triart PFP (φ4.6×250 mm, eluted with 70% MeOH)] to afford compound **27** (4.6 mg). Fr. 3-5-3-4-4 (11.5 mg) was further separated via preparative HPLC [Cosmosil AR-II C_18_ (φ10×250 mm, eluted with 70% MeOH)] to purify compound **25** (5.9 mg) and **26** (4.5 mg). Fr. 3-5-3-4-5 (19.7 mg) was purified via two preparative HPLC columns [Cosmosil π Nap [φ4.6×250 mm, eluted with 60% MeOH) and Triart PFP (φ4.6×250 mm, eluted with 70% MeOH)] to give compound **27** (4.6 mg). Fr. 3-5-3-4-6 (37.3 mg) was subjected to preparative HPLC column (Cosmosil π Nap [φ10×250 mm, eluted with 80% MeOH)] to afford compound **28** (17.1 mg). Fr. 3-5-5 (200 mg) was loaded onto SiO_2_ (φ10×130 mm, using CHCl_3_: MeOH: H_2_O = 9:1:0.1 (*v/v*)) to yield 6 fractions (frs. 3-5-5-1 to 3-5-5-6). Then two fractions (frs. 3-5-5-4 and -5) was purified via preparative HPLC (Cosmosil C_18_ AR-II (φ10×250 mm, eluted with 70% MeOH) to give compound **29** (1.9 mg), **30** (3.3 mg), respectively. A fraction 3-5-5-6 was purified via two preparative HPLC columns [Cosmosil C_18_ AR-II (φ10×250 mm), eluted with 70% MeOH and X-Bridge Prep. C_18_ (φ10×250 mm, eluted with 65% MeOH)] to afford compound **31** (4.0 mg), **32** (14.5 mg), and **33** (6.3 mg). Fr. 3-6 (12.2 g) was subjected to Sephadex LH-20 (φ20×1000 mm, eluted with MeOH) to yield 5 fractions (frs. 3-6-1 to 3-6-5). As the next step, fr. 3-6-4 (188 mg) was loaded onto μ-Bonda Pak C_18_ column chromatography (φ25×200 mm) and eluted with an H_2_O–MeOH gradient (70, 80, 90 % MeOH; 135 mL of each gradient solution) to afford 8 fractions (frs. 3-6-4-1 to 3-6-4-8). Fr. 3-6-4-4 (15.6 mg) was purified via preparative HPLC [Cosmosil C_18_ AR-II (φ10×250 mm, 80%MeOH)] to give compound **34** (2.8 mg). Fr. 3-6-4-5 (37.2 mg) was separated using two preparative HPLC columns [Cosmosil C_18_ AR-II (φ10×250 mm, eluted with 80%MeOH and Triart PFP (φ4.6×250 mm, eluted with 75% MeOH) to afford compound **35** (2.5 mg). Fr. 3-6-4-6 (23.4 mg) was subjected to preparative HPLC column [Cosmosil C_18_ AR-II (φ10×250 mm, eluted with 85% MeOH (*v/v*))] to give compound **36** (5.9 mg). Fr. 3-6-4-7 (8.0 mg) was purified via preparative HPLC [Cosmosil C_18_ AR-II (φ10×250 mm, 80% MeOH)] to yield compound **37** (4.6 mg). Fr. 3-6-5 (123 mg) was loaded onto μ-Bonda Pak C_18_ column chromatography (φ25×200 mm) and eluted with an H_2_O–MeOH gradient (75, 80, 85, 90 % MeOH; 135 mL of each gradient solution) to afford 5 fractions (frs. 3-6-5-1 to 3-6-5-5). Fr.3-6-5-3 (43.8 mg) was purified via preparative HPLC [Cosmosil C_18_ AR-II (φ10×250 mm, 85% MeOH)] to afford compound **38** (3.7 mg), **39** (33.7 mg), and **40** (4.0 mg). A part of fr. 3-7 (4.0 g) was subjected to Sephadex LH-20 (φ20×1000 mm, eluted with MeOH) to yield 4 fractions (frs. 3-7-1 to 3-7-4). Fr. 3-7-3 (180 mg) was loaded onto μ-Bonda Pak C_18_ column chromatography (φ25× 200 mm) and eluted with an H_2_O–MeOH gradient (80, 90, 100 % MeOH; 135 mL of each gradient solution) and SiO_2_ (φ10× 80 mm, CHCl_3_: MeOH: H_2_O = 9:1:0.1 (*v/v*), preparative HPLC [Cosmosil C_18_ AR-II (φ10× 250 mm, eluted with 80% MeOH)] to afford compound **41** (0.9 mg). Fr. 3-7-4 (189 mg) was subjected to μ-Bonda Pak C_18_ column chromatography (φ25×200 mm) and eluted with an H_2_O–MeOH gradient (80, 90, 100 % MeOH; 135 mL of each gradient solution) to give 5 fractions (frs. 3-7-4-1 to 3-7-4-4). Fr. 3-7-4-2 (135.6 mg) was loaded again onto μ-Bonda Pak C_18_ column chromatography (φ25×200 mm) and eluted with an H_2_O–MeOH gradient (80, 90 % MeOH; 135 mL of each gradient solution) to give 5 fractions (frs. 3-7-4-2-1~ 3-7-4-2-5). Fr. 3-7-4-2-2 (42.2 mg) was purified via preparative HPLC [X-Bridge Prep. C_18_ (φ10×250 mm, eluted with 80% MeOH)]to afford compound **42** (28.0 mg). Fr. 3-7-4-2-5 (14.9 mg) was subjected to SiO_2_ (φ10×80 mm, CHCl_3_: MeOH: H_2_O = 9:1:0.1 (*v/v*)) and purified via preparative HPLC [Triart Phenyl (φ4.6×250 mm, eluted with 85% MeOH)] to give compound **43** (2.0 mg)

# Supplementary Figures

|  | **R_1_** | **R_2_** | **R_3_** | **R_4_** |
| --- | --- | --- | --- | --- |
| **5** (epimedoside C) | **H** | **H** | **H** | **glc** |
| **6** (cuhuoside) | **H** | **Me** | **H** | **glc^4^-glc** |
| **7** (epimedin A) | **H** | **Me** | **rha^2''^-rha** | **glc** |
| **8** (epimedin B) | **H** | **Me** | **rha^2''^-xyl** | **glc** |
| **9** (epimedin C) | **H** | **Me** | **rha^3''^-glc** | **glc** |
| **18** (epimedin I) | **H** | **Me** | **rha** | **glc** |
| **19** (sagittatoside A) | **H** | **H** | **rha^2''^-glc** | **H** |
| **20** (2”-*O*-rhamnosyl icariside II) | **H** | **Me** | **rha^2''^-rha** | **H** |
| **21** (pherodendroside) | **H** | **H** | **glc** | **H** |
| **22** (caohuoside C) | **OH** | **Me** | **rha** | **H** |
| **23** (icariside II) | **H** | **Me** | **rha** | **H** |
| **24** (icarisoside A) | **H** | **H** | **rha** | **H** |
| **30** (icariside I) | **H** | **Me** | **H** | **glc** |

**Figure S1.** Known compounds (**5**-**9** and **18**-**24**, **30**) isolated from EH.

|  | **R_1_** | **R_2_** | **R_3_** | **R_4_** | **R_5_** |
| --- | --- | --- | --- | --- | --- |
| **10** (epimedin I) | **H** | **H** | **H** | **H** | **glc** |
| **11** (epimedin K) | **Ac** | **H** | **H** | **Ac** | **glc** |
| **12** (epimedin L) | **H** | **Ac** | **H** | **Ac** | **glc** |
| **13** (caohuoside B) | **H** | **H** | **Ac** | **Ac** | **glc** |
| **14** (epimedokoreanoside I) | **H** | **H** | **H** | **Ac** | **glc** |
| **25** (korepimeoside A) | **Ac** | **H** | **H** | **Ac** | **H** |
| **26** ( korepimeoside B) | **H** | **Ac** | **H** | **Ac** | **H** |
| **27** (epimedigrandioside A) | **H** | **H** | **Ac** | **Ac** | **H** |
| **28** (korepimedoside A) | **H** | **H** | **H** | **Ac** | **H** |

**Figure S2.** Known compounds (**10**-**14** and **25**-**28**) isolated from EH.

**4** (koreanoside I)

**15** (epimedokoreanin C)

|  | **R** |
| --- | --- |
| **16** (koreanoside F) | **H** |
| **17** (koreanoside G) | **Me** |

**Figure S3.** Known compounds (**4**, **15**-**17**) isolated from EH.

|  | **R_1_** | **R_2_** | **R_3_** |
| --- | --- | --- | --- |
| **29** (8-prenyl kaempferol) | **H** | **H** | **OH** |
| **31** (8-prenyl luteolin) | **H** | **OH** | **H** |
| **37** (8,5’-diprenyl apigenin ) | **CH_2_CH=C(CH_3_)_2_** | **H** | **H** |
| **38** (broussonol D) | **CH_2_CH=C(CH_3_)_2_** | **OH** | **OH** |
| **39** (epimedokoreanin B) | **CH_2_CH=C(CH_3_)_2_** | **OH** | **H** |

**Figure S4.** Known compounds (**29**, **31**, **37-39**) isolated from EH.

**33** (epicornunin F)

**32** (epicornunin B)

|  | **R** |
| --- | --- |
| **34** (gaocaonin E) | **OH** |
| **35** (euchrestaflavanone A) | **H** |

**36** (epimedonin C)

**Figure S5.** Known compounds (**32**-**36**) isolated from EH.

**40** (epimedonin E)

|  | **R_1_** | **R_2_** | **R_3_** |
| --- | --- | --- | --- |
| **41** (4’-*O*-methyl limonianin) | **H** | **Me** | **H** |
| **42** (limonianin) | **H** | **H** | **H** |
| **43** (epimedonin F) | **CH_2_CH=C(CH_3_)_2_** | **H** | **H** |

**Figure S6.** Known compounds (**40**-**43**) isolated from EH.


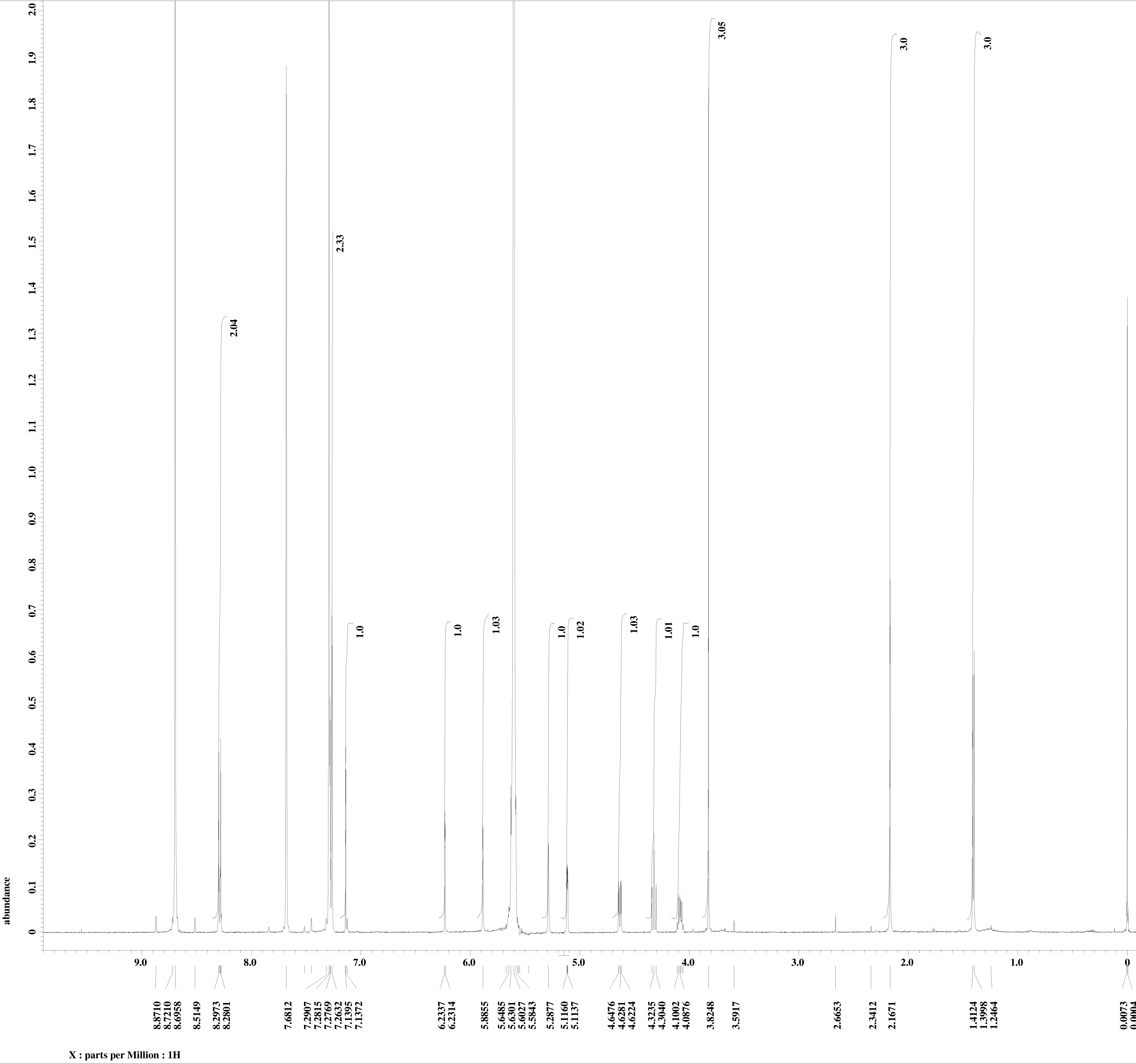


**Figure S7.** ^1^H NMR spectrum of **1** (in Pyridine-*d_5_*, 500 MHz)


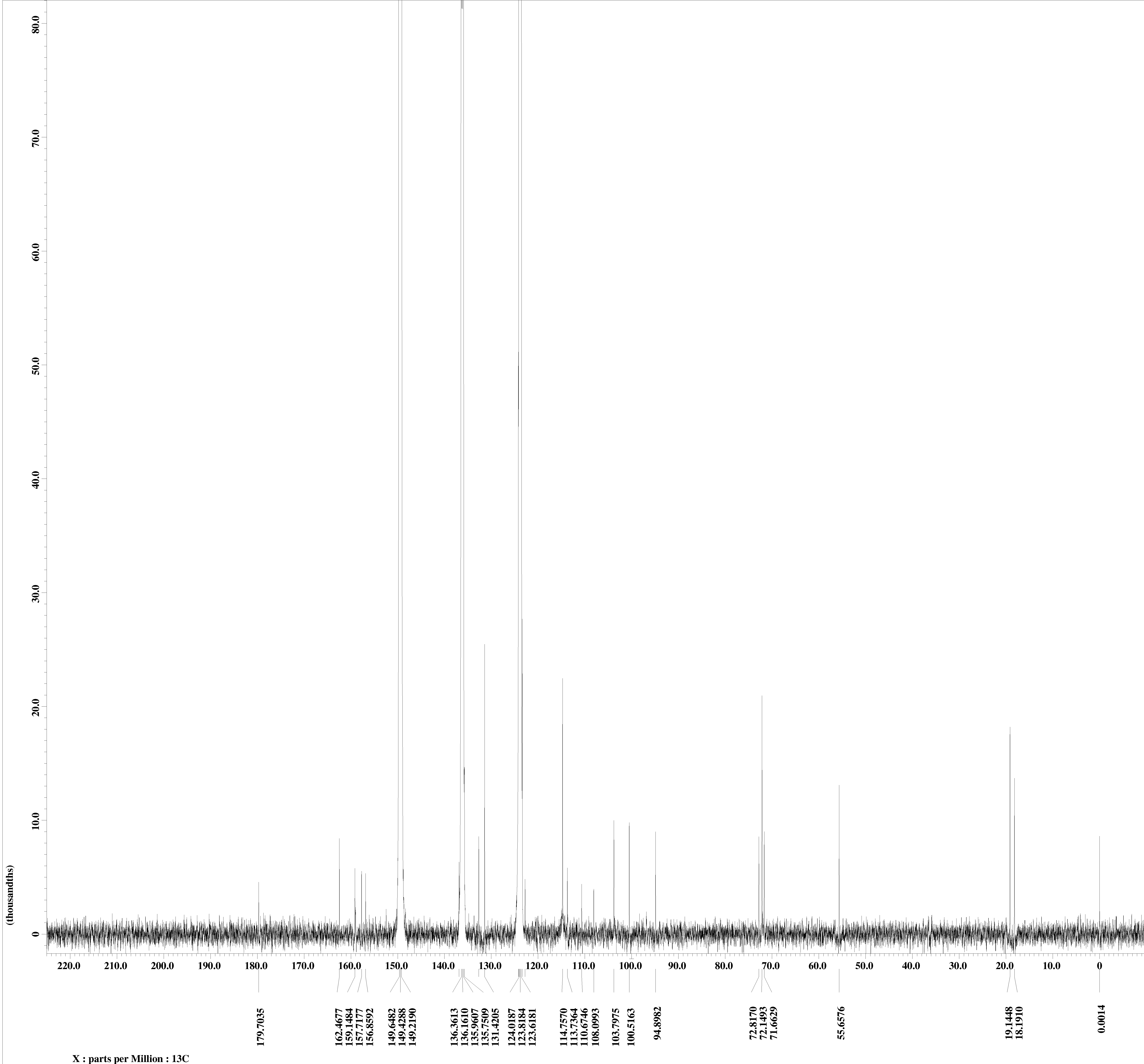


**Figure S8.** ^13^C NMR spectra of **1** (in Pyridine-*d_5_*, 125 MHz)


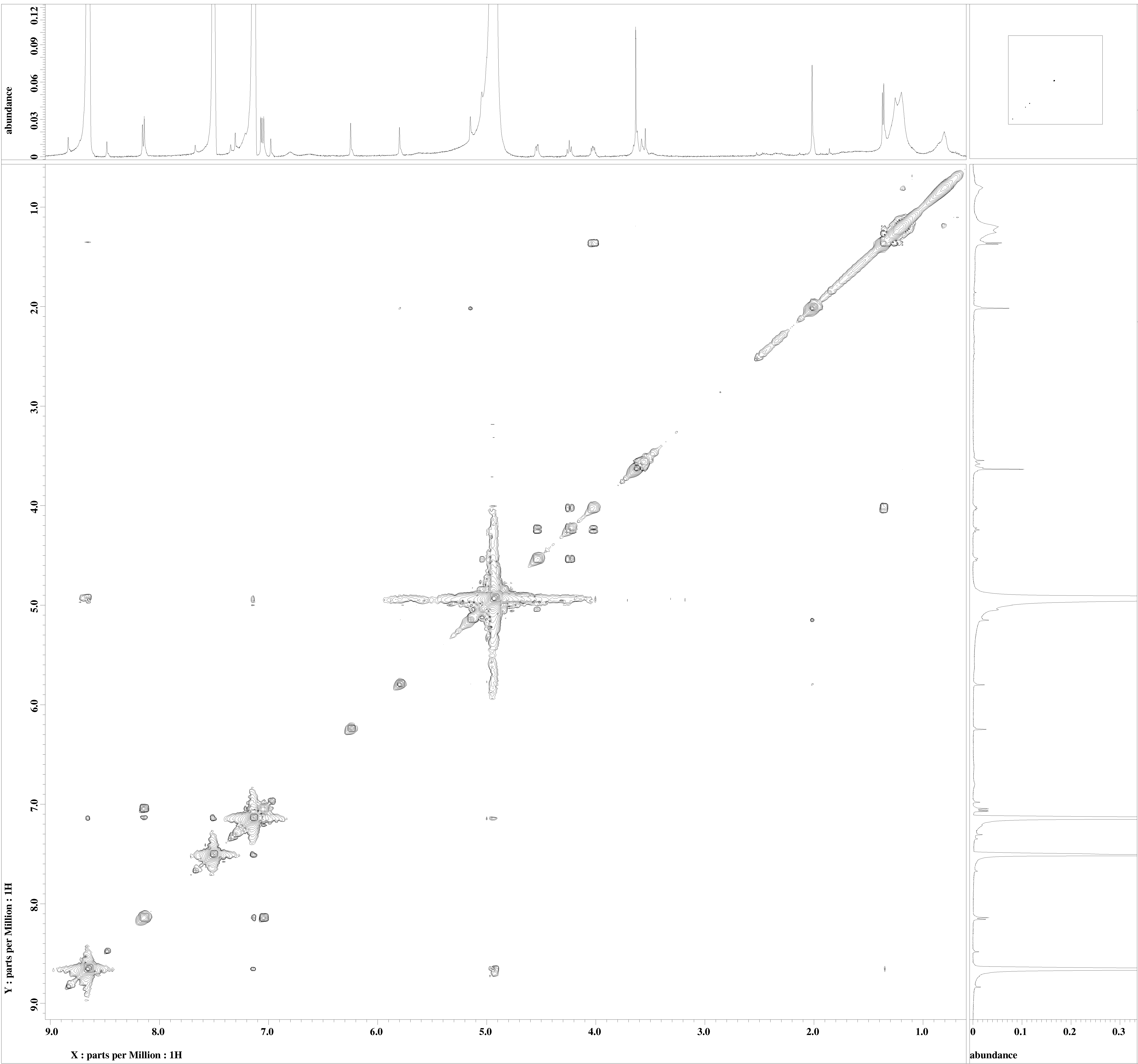


**Figure S9.** ^1^H-^1^H COSY spectrum of **1** (in Pyridine-*d_5_*, 500 MHz)


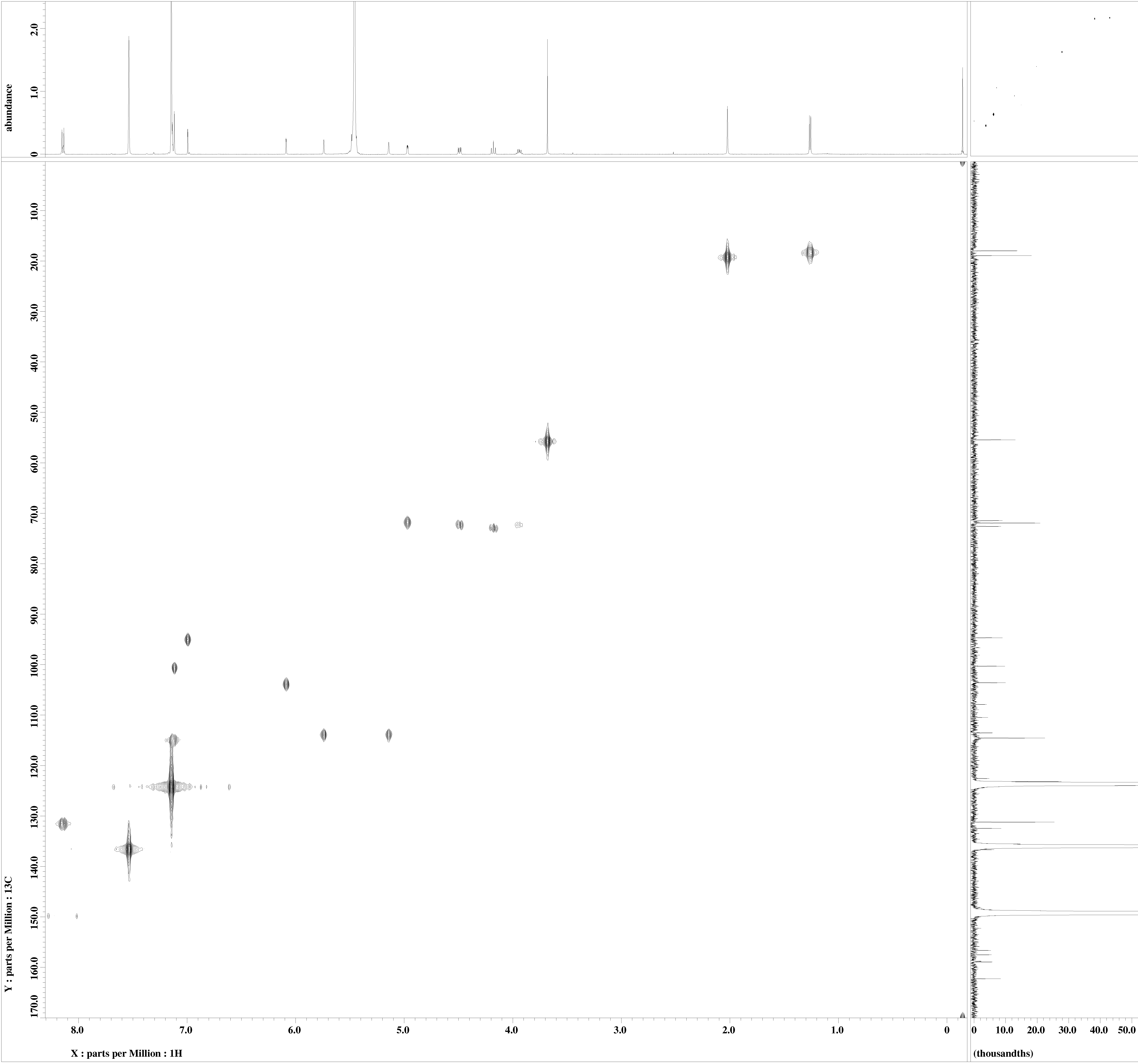


**Figure S10.** HMQC spectrum of **1** (in Pyridine-*d_5_*, 500 MHz)


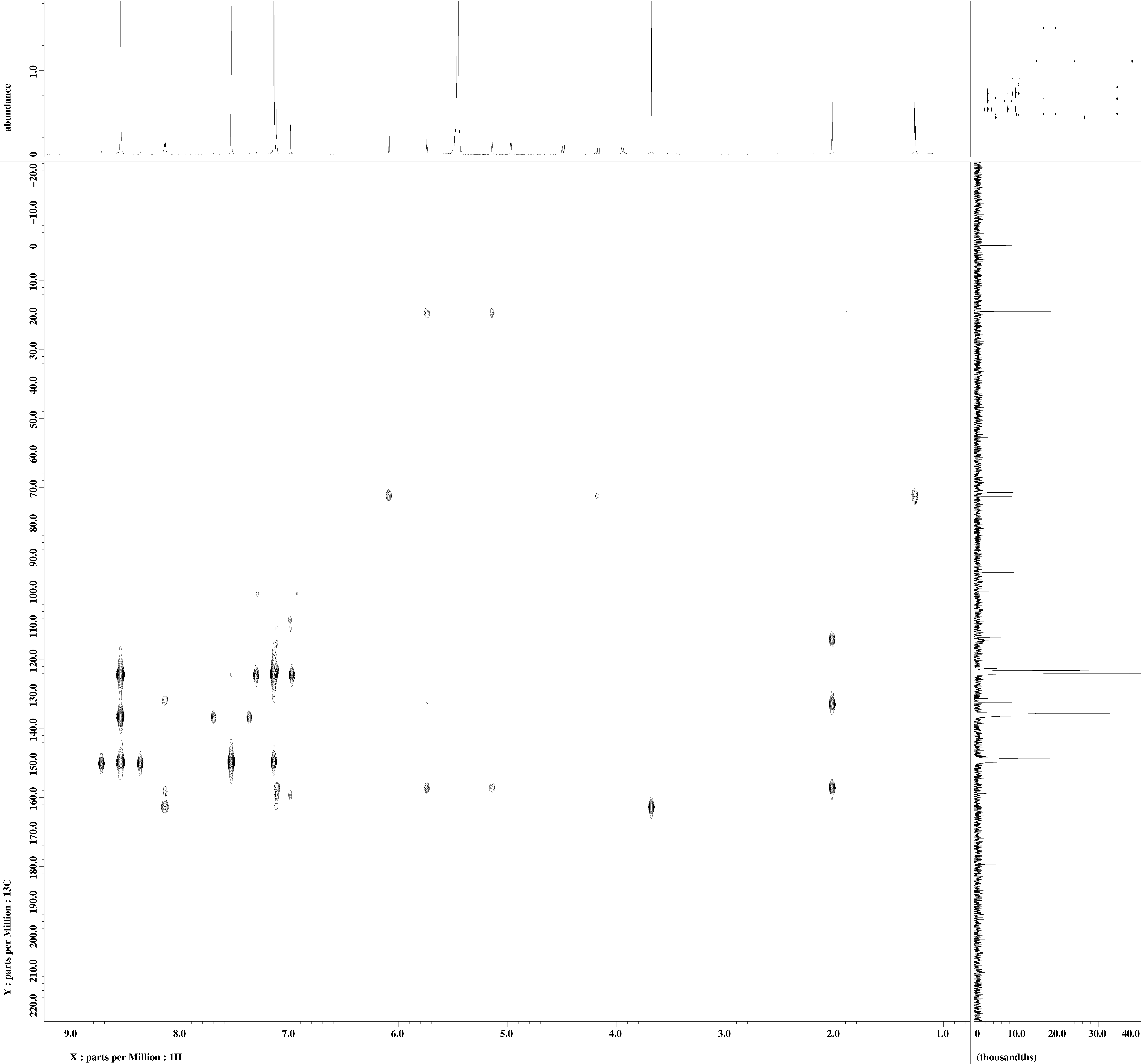


**Figure S11.** HMBC spectrum of **1** (in Pyridine-*d_5_*, 500 MHz)


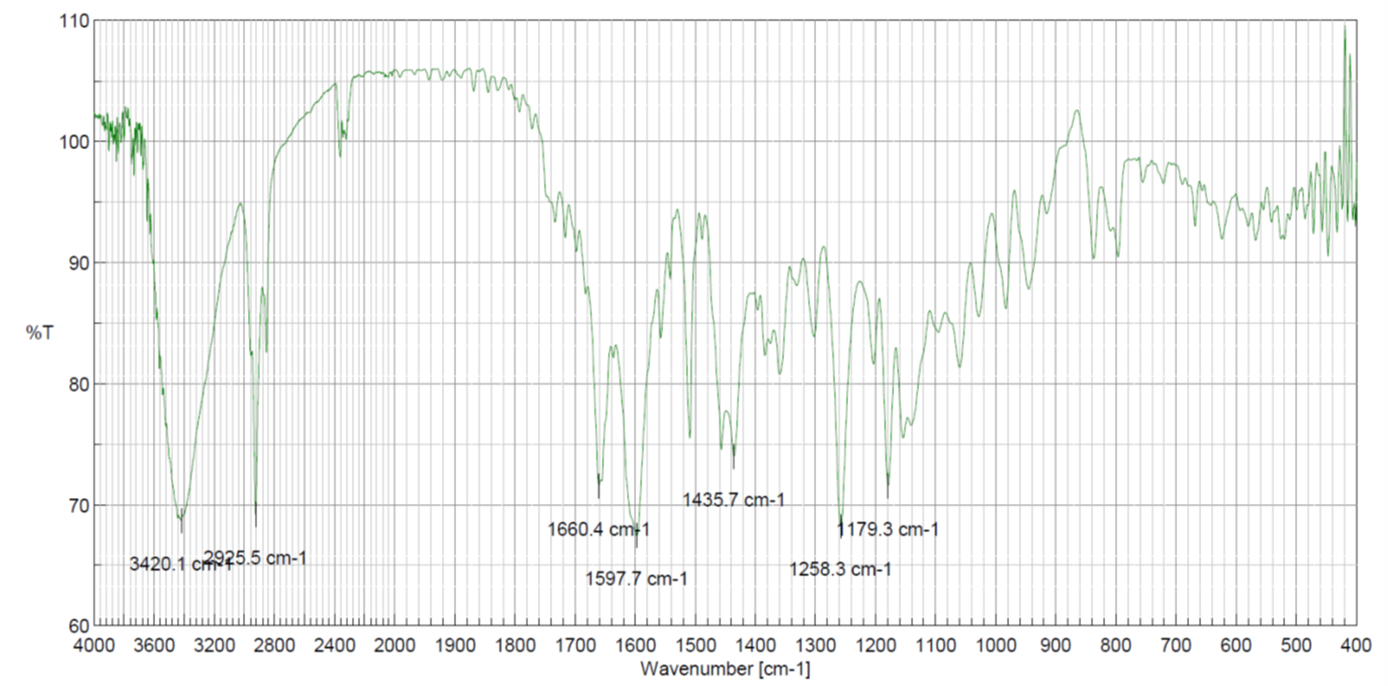


**Figure S12.** FT-IR spectrum of **1**


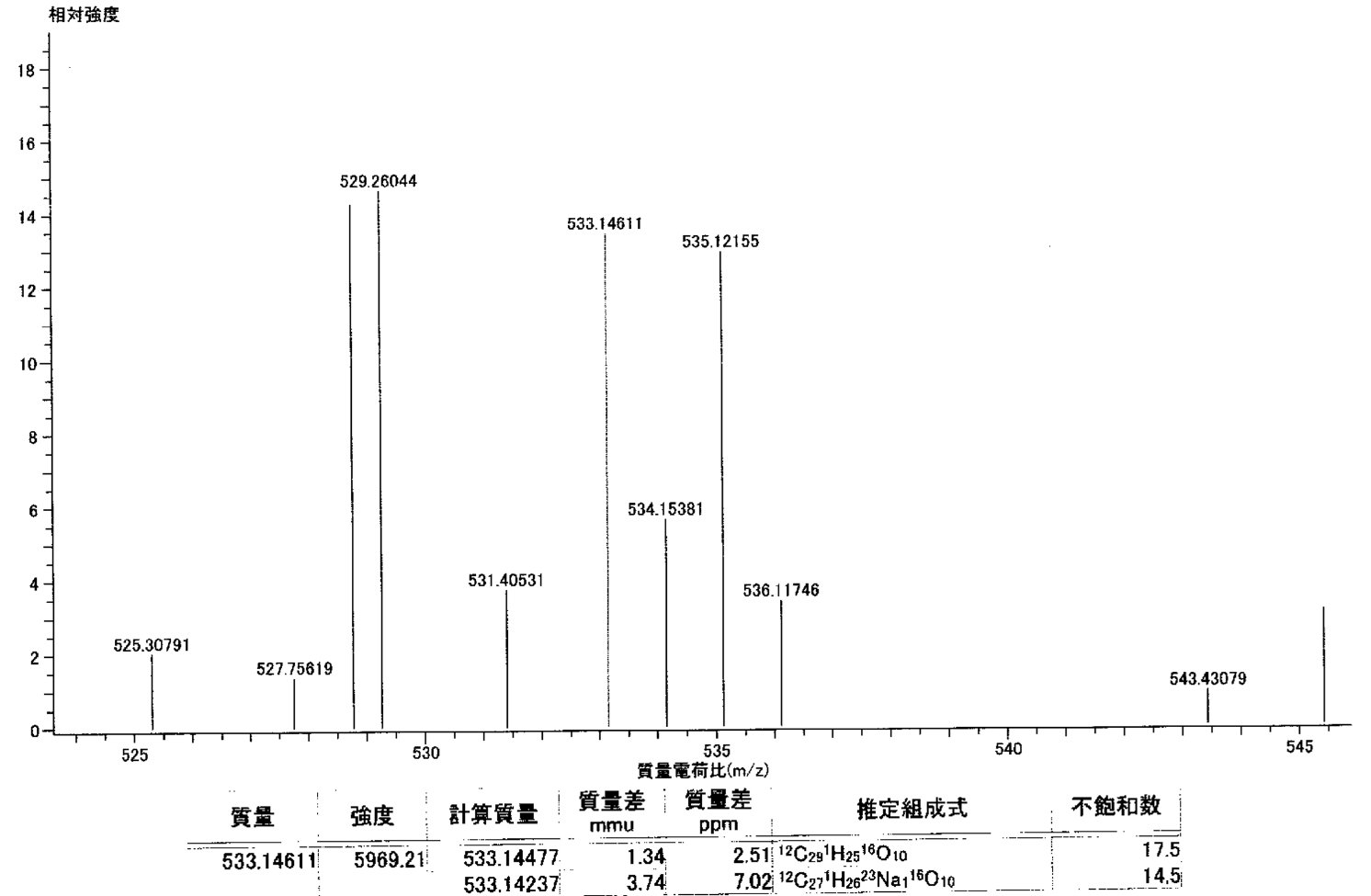


**Figure S13.** HR-positive-ion ESI TOF-MS data of **1**
